# Invasive and Non-Invasive Remote Patient Monitoring Devices for Heart Failure: A Comparative Review of Technical Maturity and Clinical Readiness

**DOI:** 10.3390/s25206453

**Published:** 2025-10-18

**Authors:** Ivan Luque, Mar Gadea, Anna Comas, Laura Becerra-Fajardo, Javier Colás, Antoni Ivorra

**Affiliations:** 1 Biomedical Electronics Research Group, Department of Engineering, Universitat Pompeu Fabra, 08018 Barcelona, Spain; ivan.luque@upf.edu (I.L.); mar.gadea@upf.edu (M.G.); anna.comas@upf.edu (A.C.); laura.becerra@upf.edu (L.B.-F.); jcolas@valorensalud.es (J.C.); 2 Institute for Healthcare Management, Business and Law School, Esade Ramon Llull University, 08034 Barcelona, Spain; 3 Cluster for Technological Innovation and Talent in Biomedical Technologies and Biotechnology of the Community of Madrid, 28003 Madrid, Spain; 4 Serra Húnter Fellow Programme, Department of Engineering, Universitat Pompeu Fabra, 08018 Barcelona, Spain

**Keywords:** heart failure, remote monitoring, remote patient monitoring, remote patient management, sensors, invasive, non-invasive, telehealth

## Abstract

Heart failure (HF) represents a growing public health concern, driven by rising prevalence and the challenge of frequent, costly (re-)hospitalizations from decompensation. To address these, HF management has progressed towards incorporating devices for remote patient monitoring (RPM), with most being focused on identifying decompensation and providing timely, tailored pharmacological interventions. To date, the pool of devices has enlarged substantially, forming a spectrum of invasive and non-invasive options whose clinical adoption potential is yet to be determined. This review summarizes existing devices for RPM in HF care, with a major focus on technical characteristics and potential clinical efficacy. To unify the two traditionally separated groups, we re-classify the sampled devices in a single taxonomical dimension, the physical location of the sensing element(s), and objectively assess their current development state using the Medical Device Readiness Level, a metric that merges technical and clinical perspectives. Furthermore, we outline additional evaluative metrics within two complementary dimensions, focused on process efficiency and patient outcomes, ultimately offering a structured framework to evaluate clinical adoption.

## 1. Introduction

Heart failure (HF) represents a growing public health concern and is the leading cause of hospitalization in patients older than 65. Its rising prevalence is largely attributable to advances in diagnostic techniques and the widespread use of life-prolonging therapies for various comorbid conditions, which have enabled patients to survive longer [1].

At present, the clinical burden of HF is immense, with over 6 million cases in the U.S. alone and an annual cost exceeding USD 30 billion. With an aging population and rising rates of comorbidities, HF is projected to affect 1 in every 33 Americans by 2030, with an associated annual cost of USD 70 billion [2]. From these, recurrent, unplanned hospitalizations represent the largest component, with costs ranging between USD 10,737 and USD 17,830 per hospitalization [3].

Clinically, HF hospitalizations are characterized by signs of fluid overload, such as orthopnea, peripheral edema, and weight gain, and symptoms of low cardiac output (CO) and hypoperfusion, like fatigue, dyspnea, confusion, or hypotension. At a fundamental level, these signs and symptoms reflect a shift toward cardiac dysfunction, in a heart with reduced baseline function [4].

The different mechanisms underlying cardiac dysfunction manifest as distinct HF phenotypes, commonly classified by left ventricular ejection fraction into preserved (HFpEF, ≥50%), mildly reduced (HFmrEF, 40–49%), and reduced (HFrEF, <40%). Though diverse in their mechanisms, these different subtypes produce similar downstream events and symptomatology [5].

**Figure 1:**
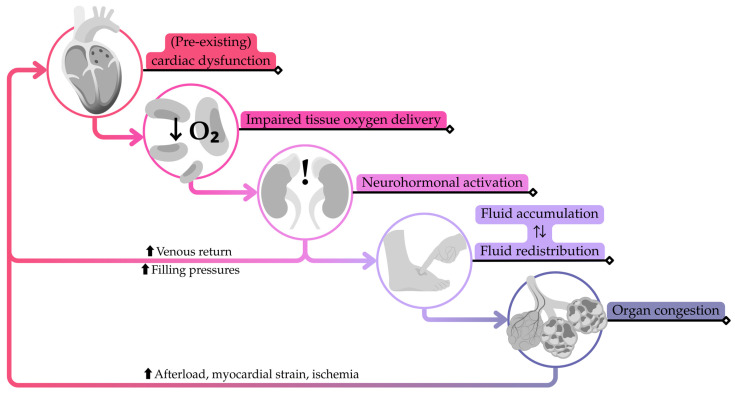
Simplified pathophysiological cascade of heart failure decompensation following an initial trigger causing a slight decline in cardiac output. Adapted from W.M. Raffaelo et al. [4].

Under normal physiological conditions, a series of compensatory neurohormonal mechanisms, including the renin–angiotensin–aldosterone system and the sympathetic nervous system, help reach an adequate cardiac function, preserving CO and organ perfusion. For patients with chronic HF under treatment, however, these same processes may promote fluid retention and increase cardiac burden, creating a maladaptive cycle. Triggers such as high sodium intake or poor medication adherence can overwhelm these responses, precipitating the functional deterioration. This process is referred to as HF decompensation (Figure 1) [4,6].

During decompensation, fluid accumulates and redistributes, leading to symptoms that often require urgent medical attention. These clinical manifestations are preceded by consistent physiological changes, such as a rise in cardiac filling pressures, an increase in circulating blood volume, and an accumulation of extravascular fluid, which are usually unnoticed [7].

Considering all this, and mainly driven by the need to reduce costs, HF management has progressed towards identifying HF worsening remotely, and providing timely, tailored pharmacological interventions in a data-driven loop. This is a proactive strategy that aims to leverage the high patient variability, alleviate the burden on healthcare centers, and turn patients into active players of their own care.

Multiple attempts at remote patient monitoring (RPM) through the use of traditional, patient-reported data (weights, vital signs, and visible symptoms like peripheral edema or fatigue) have continuously failed to demonstrate benefits through clinical trials and experience [8,9]. In a quest to find the right tool and close this gap, a series of devices, with smarter and superior sensing capabilities, have been developed in the past three decades; a process that has dramatically accelerated since 2014, after the approval by the Food and Drug Administration (FDA) of the invasive CardioMEMS™ HF System (formerly St. Jude Medical, Inc., now Abbott, Illinois City, IL, USA). Following this, the vast majority of RPM systems for HF have primarily aimed at preventing decompensations, while others are being developed with additional objectives, such as facilitating long-term management through continuous monitoring, supporting medication adherence, or enabling precise titration.

As a result, the pool of devices has enlarged substantially to date, forming a broad spectrum of invasive and non-invasive options. In line with this, the latest 2021 European Society of Cardiology guidelines provide only selective recommendations for both groups, namely a Class IIb recommendation meaning their use “may be considered” [10]. Since their publication, however, important advances have taken place, prompting more specific consensus statements (most notably, the 2025 Heart Failure Association of the European Society of Cardiology’s clinical consensus regarding some invasive devices [11]). These developments underscore the fast-paced and dynamic nature of the field, in which different devices are at distinct stages of development and therefore require a careful and multi-faceted assessment to fully understand their clinical adoption potential.

This review summarizes existing devices for RPM in HF care, reporting their core technological aspects, supporting evidence, future directions, and current challenges. To that end, we compare key devices retrieved from the classical invasive and non-invasive domains. However, because this categorization groups together technologies that differ markedly (e.g., intracardiac and subcutaneous devices), its potential to underscore intra-group differences is rather limited. To provide a more comprehensive review, we re-classify these devices in a single taxonomical dimension, namely the physical location of the sensing element(s) (Table 1). This framework unifies invasive and non-invasive devices under one scheme, making sub-group differences clearer and helping highlight trends, gaps, and opportunities. The devices selected for this review and their sub-groups are collected in Table 2. Note that devices with the sole purpose of HF therapy (e.g., baroreflex stimulators) or which are officially discontinued were not considered during sampling.

To objectively assess the current state of these devices, we further characterize them using the Medical Device Readiness Level (MDRL), as proposed by RR. Seva et al. [12]. Invasive devices with MDRL < 5 (not included) and non-invasive devices with MDRL < 8 (not included) are not extensively described here, but are included in Supplementary Materials.

Finally, we outline additional evaluative metrics within two complementary dimensions: one focused on improving process efficiency and the other on enhancing patient outcomes. Although a detailed discussion is beyond the scope of this review, we do provide an application example for one of the presented devices (the CardioMEMS™ HF System), in hope that the proposed dimensions will help policymakers, clinicians, and researchers to compare device strengths and limitations, contextualize MDRL, and estimate adoption potential in real-world practice in the future.

To balance accessibility for a general readership with value for expert readers, we have structured the remaining part of our review in a layered manner (i.e., sections for breadth, sub-sections for depth). The structure is as follows:

(i) Each device sub-group (Table 1) is presented in a dedicated section;
(ii) Each section begins with an executive summary describing fundamental concepts, highlighting common trends, and listing the sampled devices;
(iii) Detailed sub-sections provide technical and clinical/scientific descriptions of individual devices;
(iv) A summary section (Section 9) synthesizes technical features and clinical evidence from reviewed devices;
(v) Final sections (Section 10 and Section 11) introduce and employ evaluative domains based on objective metrics (i.e., the MDRL and the clinical adoption metrics).

## 2. Intracardiac Devices

Changes in cardiac filling pressures are among the earliest physiological parameters anticipating HF worsening, often occurring weeks before hospitalization [6]. As these signals offer unmatched insights into the dynamics of cardiac dysfunction, it is no coincidence that the beginnings of invasive RPM, back in the late 1990s, originated within the heart, with the introduction of the Chronicle^®^ (Medtronic, Inc., Minneapolis, MN, USA).

Inspired by contemporary devices, the Chronicle^®^ employed a pacemaker-type design, with an 11 Fr lead deployed in the right ventricle where pressure and oxygen saturation could be measured [13]. Although promising results in chronic preclinical studies supported its safety and accuracy, the Chronicle^®^ failed to demonstrate hospitalization reduction in its first large clinical study, the COMPASS-HF (NCT00643279, *n* = 277), initiated in 2003 [14]. As a result, the device did not obtain FDA approval and was eventually discontinued.

**Figure 2:**
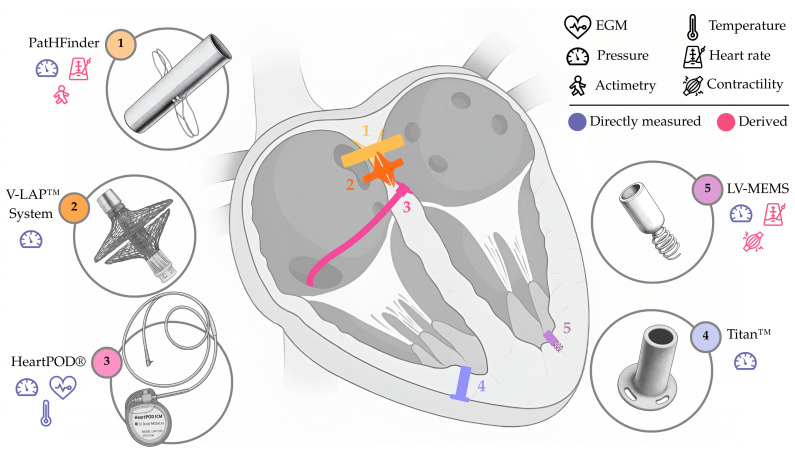
Appearance and approximate location of the sensing elements in systems designed to monitor intracardiac parameters. Directly measured and derived parameters are indicated. Not to scale. Only for illustrative purposes.

Far from discouraging innovation, the pioneering Chronicle^®^ experience served to refine trial design and inspired further development of invasive RPM devices. Modern successors still regard intracardiac sensing as core RPM strategy for RPM. These include **(i)** the HeartPOD^®^, a pacemaker-like device, with a sensing lead (element 3, Figure 2) designed for left atrial pressure (LAP) monitoring; **(ii)** the V-LAP™ System, based on a transseptal implant (element 2, Figure 2) with a focus on LAP monitoring; **(iii)** the Titan™, based on a transmyocardial implant (element 4, Figure 2) compatible with all four heart chambers, primarily for left ventricular pressure (LVP) or LAP monitoring; **(iv)** the LV-MEMS, based on a left ventricular anchored implant (element 5, Figure 2) focused on LVP monitoring; and **(v)** the PatHFinder, based on a battery-operated transseptal implant (element 1, Figure 2) for bi-atrial pressure monitoring.

Despite these advances, two decades of experience after the Chronicle^®^ demonstrate that measuring in the intracardiac domain remains a powerful yet challenging task, often limited by the complexities of access and the continuous questioning of long-term safety.

### 2.1. HeartPOD^®^ (Abbott, Illinois City, IL, USA)

Following the steps of the Chronicle^®^, the HeartPOD^®^ (formerly St. Jude Medical, Inc., now Abbott, Illinois City, IL, USA) is centered around LAP monitoring and consists of three main, modular parts: an implantable sensor module (i.e., a sensing lead; element 3, Figure 2); a subcutaneous communication module; and a patient module. Implantation begins with a right heart catheterization via femoral access under echocardiographic guidance. Using an 11 Fr sheath, a transseptal puncture is performed in the interatrial septum. The tip of the sensing lead, equipped with folded anchors, is then advanced across the septum. Once delivered, the anchors secure the lead to the septum, exposing the tip to the left atrium. In recent implantations, the proximal part of the lead is repositioned to exit via a superior atrial vein (right or left axillary, or subclavian), and then connected to the communication module (~33 mm diameter), secured in a subcutaneous pocket [15,16].

The sensing lead comprises a 3 mm × 7 mm hermetic module with a titanium pressure sensing membrane, dedicated circuitry, and folding nitinol anchors. The patient advisory module uses a handheld device, positioned in contact with the skin, over the subcutaneous module, to power and communicate via radiofrequency.

Patients perform daily 15 s measurements after resting for 3 min in a recumbent position, which allows the device to report LAP, temperature, and an intracardiac electrogram. Patients may also log symptoms, daily weight, and blood pressure to complement the data [15,16,17].

After demonstrating in vivo that LAP measurements correlated with catheter counterparts (r = 0.95) (*n* = 8) [15], the HeartPOD^®^ proceeded to its first in-human trial, HOMEOSTASIS (NCT00547729, *n* = 40, 23% HFpEF/HFmrEF, 77% HFrEF) [18]. The study confirmed its feasibility and accuracy, and became the first to successfully execute physician-directed patient self-management, based on individualized LAP thresholds, for titration of diuretics and vasodilators. This trial also reported a decline in HF hospitalizations and an improvement in functional status, which encouraged a larger follow-up trial. The LAPTOP-HF (NCT01121107, *n* = 730) [16] became the next natural step in validating the device and its LAP-based self-management strategy, aiming to show safety and reduced relative risk of hospitalization at 1 year post-implantation. However, excessive complications during implantation halted enrollment to the trial, leaving a sample size of *n* = 486 (~75% HFpEF/HFmrEF, ~25% HFrEF), and compromising the statistical power to claim clinical effectiveness. Patients who remained in follow-up showed that LAP-directed therapy achieved a 41% hospitalization reduction at 1 year post-implantation [19]. More recent data also showed a significant 44% mortality reduction in LAPTOP-HF at 2 years post-implantation [20].

The HeartPOD^®^ has not pursued regulatory approval after the LAPTOP-HF experience, nor has it proceeded with further trialing, effectively freezing efforts in development and commercialization. Despite obvious challenges (e.g., periprocedural safety), the device still preserves historical relevance, mostly as the first serious effort at self-management in HF, and as a clear demonstration of the potential value of intracardiac sensing.

### 2.2. V-LAP™ System (Vectorious Medical Technologies, Ltd., Tel Aviv, Israel)

The V-LAP™ System (Vectorious Medical Technologies, Ltd., Tel Aviv, Israel) uses a wireless, batteryless, minimally invasive sensor implanted at the interatrial septum, representing the leadless iteration of the earlier HeartPOD^®^ device. Implantation requires a dedicated, 12 Fr delivery system and the help of angiographic and echocardiographic guidance. The implant (element 2, Figure 2), measuring <18 mm in length and 3.8 mm in diameter, houses microelectromechanical pressure transducers capable of measuring LAP.

The implant is powered with a belt-like external unit, which establishes bidirectional communications via radiofrequency, and transmits the LAP data to a cloud-based platform through Wi-Fi or cellular connectivity [21]. The patient places the external unit over clothing, around the chest, and performs a daily measurement at rest, lasting 1–3 min. The transmitted data are accessible only to the treating physician.

The V-LAP™ System was demonstrated to be safe and accurate in early studies, showing strong correlation with catheter values. This included an animal study (r = 0.95) (*n* = 10, ovine) [21], as well the first in-human study (Lin’s concordance = 0.79), called VECTOR-HF (NCT03775161, *n* = 30, ~8% HFpEF/HFmrEF, ~92% HFrEF) [22,23]. Furthermore, a pilot study introduced ambulatory physician-directed patient self-management in patients enrolled in VECTOR-HF and VECTOR-HF IIa (a study in Tbilisi, Georgia), where they self-adjusted their diuretic dosage through physician-prescribed thresholds. While the study offers limited evidence, the findings suggest that this strategy could lead to high patient adherence and improved clinical outcomes while reducing the clinicians’ burden [24].

The V-LAP™ System, which received Breakthrough Device Designation by the FDA in 2020, holds great potential as an invasive option for LAP-based RPM. The strength of the device lies in its superior sensitivity when compared with intravascular counterparts for pulmonary artery pressure (PAP) measurements, which have dominated the invasive space for the past decade. This is particularly true for certain clinical scenarios including, but not limited to, atrial arrhythmias, functional mitral regurgitation, and increased pulmonary resistance, all of which may act as confounders for PAP rise. On the other end, the implant’s placement can obstruct the interatrial septum and potentially limit future transseptal interventions, such as mitral valve procedures or left atrial appendage closure.

### 2.3. Titan™ (Integrated Sensing Systems, Inc., Ypsilanti, MI, USA)

The Titan™ (Integrated Sensing Systems, Inc., Ypsilanti, MI, USA) revolves around a wireless, batteryless sensor implanted across the myocardium of one of the left heart chambers (i.e., left ventricle or left atrium), following an ultrasound-guided incision in the cardiac wall, and the help of a 14 Fr dilator. The implant (element 4, Figure 2) is composed of a housing with four flange holes at the proximal end, which help in suturing the implant to the epicardium. The housing includes a cylindrical sensing probe, with a distal microelectromechanical pressure sensor, an electronic circuit, and a telemetry antenna, which protrudes inside the endocardium. The implant is available in four different lengths, from 18 to 30 mm, and has a diameter of 3.7 mm. The system incorporates an external transceiver unit with a handheld antenna that powers the implant through electromagnetic induction and receives the pressure measurements via radiofrequency. Finally, these are sent to a computer via USB.

After initial animal studies, including a 4-week-long trial (*n* = 4, canine), the Titan™ was demonstrated to be safe, with no thromboembolic complications [25]. In the first in-human study (*n* = 40), the device continued to show safety for more than a year, with accurate pressure measurements comparable to catheter ones (r = 0.96) [26]. Though the device was implanted in the left heart for human studies, early pilot and animal studies demonstrated that it could be implanted in all four heart chambers, the right atrial appendage, and the descending aorta [26,27].

The potential of the Titan™ lies in its versatility in documented implantation locations, as well as in demonstrated usefulness for patients undergoing left ventricular support, as seen in a series of case reports (*n* = 4) following the first in-human study [28]. Nevertheless, implantation of the Titan™ requires an open chest procedure, and safety beyond 1 year post-implantation is still to be determined in a large cohort of patients. Whether tailored medication following Titan™ measurements can reduce HF hospitalizations is also yet to be determined.

## 3. Intravascular Devices

**Figure 3:**
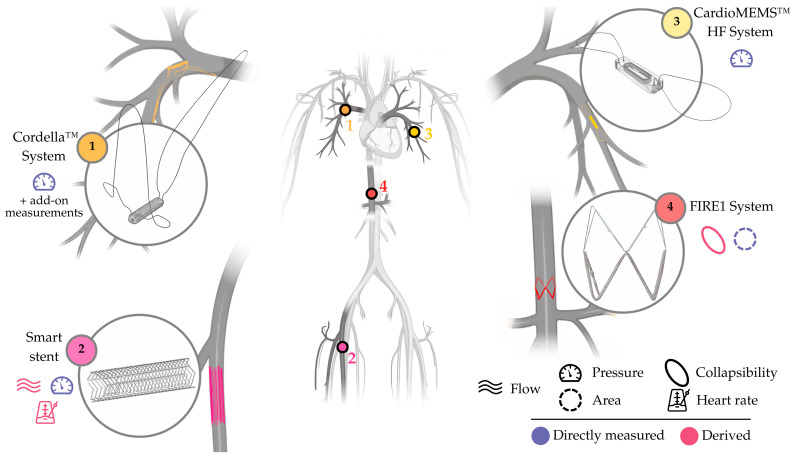
Appearance and approximate location of the sensing elements in systems designed to monitor intravascular parameters. Directly measured and derived parameters are indicated. Not to scale. Only for illustrative purposes.

The true breakthrough for invasive devices came with the CardioMEMS™ HF System (element 3, Figure 3), approved by the FDA in 2014 and still commercially available to date. The regulatory triumph crystallized the value of RPM, accelerating development of other invasive and non-invasive devices. The CardioMEMS™ HF System adequately identified the inherent risks of intracardiac monitoring, and the value of PAP as an adequate surrogate for cardiac filling pressures (arguably the actual drivers of cardiac dysfunction). Results from early clinical trials of the CardioMEMS™ HF System, namely CHAMPION (NCT00531661) and GUIDE-HF (NCT03387813), shaped the considerations of use for PAP-guided RPM in the U.S. and Europe, which are applicable to date. Concerns inherited from intracardiac devices, particularly about safety, were largely resolved by the excellent safety profile reported for the CardioMEMS™ HF System.

However, its success also accelerated the development of other intravascular devices. These positioned their value through alternative implantation sites, additional physiological parameters, new modes of data interaction, or a combination of these approaches.

Taken together, these devices form a landscape that can be simplified as follows: **(i)** the aforementioned CardioMEMS™ HF System, based on a pulmonary artery implant (element 3, Figure 3), capable of measuring PAP; **(ii)** the Cordella™ System, also based on a pulmonary artery implant (element 1, Figure 3), primarily intended for PAP monitoring but complemented with external add-on measurements; **(iii)** the FIRE1 System (now NORM™ System), based on an inferior vena cava (IVC) implant (element 4, Figure 3), capable of measuring IVC area; and **(iv)** smart stents, such as that developed by R. Herbert et al. [29] (element 2, Figure 3).

Despite their promise, intravascular devices face common technical challenges, including long-term calibration issues and the need for high patient adherence to operate external units.

### 3.1. CardioMEMS™ HF System (Abbott, Illinois City, IL, USA)

The CardioMEMS™ HF System (formerly St Jude Medical, Ltd., now Abbott, Illinois City, IL, USA) is based on a wireless, batteryless, passive sensor (element 3, Figure 3) implanted in an inferior, lateral branch of the left pulmonary artery measuring 7–15 mm. Implantation is performed under fluoroscopy after a right heart catheterization via femoral venous access. An 8 Fr sheath is upsized to 12 Fr, through which the device is advanced. Once deployed, two nitinol loops anchor the implant to the vessel wall, and fluoroscopy confirms its location.

The rigid part of the implant measures 15 mm × 3 mm × 2 mm and consists of a coil and a capacitor, forming a resonant circuit that responds to PAP. These are encased within a hermetically sealed, fused silica capsule coated with medical-grade silicone. The nitinol loops, 10 mm in length, are located at both ends of the case and provide mechanical fixation inside the vessel lumen. The implant is interrogated using a pillow-shaped external system, placed against the patient’s back in a supine position.

Collected data are later uploaded to a secure cloud-based platform for review, accessible only to the treating physician. Patients are instructed to take a daily PAP measurement, which takes 2–5 min [30,31,32].

The CardioMEMS™ HF System was validated in the pivotal CHAMPION clinical trial (NCT00531661, *n* = 550, ~21.5% HFpEF/HFmrEF, ~78.5% HFrEF), which met all primary and secondary endpoints. These included a reduction in the rate of HF hospitalizations by 28%, thanks to PAP-directed medication [33]. The positive results in safety and effectiveness led to Premarket Approval by the FDA in 2014. However, approval of the device for clinical use was contingent upon results of the CardioMEMS™ HF System Post Approval Study (NCT02279888, *n* = 1200, 30% HFpEF, 17% HFmrEF, 53% HFrEF). This study incorporated clinical sub-groups underrepresented in the CHAMPION, and addressed trial design concerns regarding nurse involvement in the treatment arm. Once again, results showed significant hospitalization reduction (consistent across sex, race, and clinical phenotype), with very low complication (0.4%) and sensor failure (0.1%) rates [34]. Evidence was reinforced by GUIDE-HF (NCT03387813, *n* = 1000, 40% HFpEF, 7% HFmrEF, 53% HFrEF) which, once adjusted for the impact of COVID-19, showed potential benefit for new groups of patients, leading to an expansion of indications, including patients with mild HF or elevated biomarkers without recent HF hospitalizations [35,36,37].

The CardioMEMS™ HF System received the Conformité Européenne (CE) marking in 2016, and was studied in Europe and Australia through the MEMS-HF (NCT02693691, *n* = 234, 27.7% HFpEF/HFmrEF, 72.3% HFrEF), the MONITOR-HF (NTR7673, *n* = 348, 27.9% HFpEF/HFmrEF, 72.1% HFrEF), and the COAST (NCT02954341, *n* = 321) [38,39,40]. Results from these trials confirmed that the device was safe and feasible, aggregating evidence across multiple healthcare systems. HF hospitalizations were reduced by 44% (MONITOR-HF) and up to 69% (COAST) at 1 year post-implantation. Recent post-market surveillance data has further stressed the safety of the device, with a mean 1.8% adverse event rate (as of early 2021, historical *n* = 20,000) [41], although recalibration requirements have been more frequent than anticipated [42].

Ongoing trials of the CardioMEMS™ HF System seek to explore the performance of the device for different follow-up strategies, including HF nurse-led management (PASSPORT-HF [43], NCT04398654) and HF nurse-directed self-management (SELFIe-HF, NCT04441203), as well as for specific clinical sub-groups. Sub-groups of interest include advanced HF (RH-SAINTS-B, NCT05284955) and cardiogenic shock (HALO-Shock, NCT04419480).

Sustained evidence on safety and efficacy, replicable across different geographical locations and healthcare systems, place the CardioMEMS™ HF System at the forefront of invasive RPM. Despite its many successes, the device has still to face several challenges, including the reorganization of HF care units to accommodate RPM and the identification of optimal clinical profiles, to enhance cost-effectiveness and obtain reimbursement across all geographies [11]. The multiple ongoing clinical trials reflect the aim of finding strategies that help deal with these challenges. Furthermore, whether PAP is the best physiological parameter for guiding therapy has also been disputed by novel devices. Competing devices, such as the FIRE1 System (see below), will help clarify this question once clinical evidence becomes available.

### 3.2. Cordella™ System (Edwards Lifesciences Corp., Irvine, CA, USA)

The Cordella™ System (Endotronix, Inc., an Edwards Lifesciences Corp. company, Irvine, CA, USA) is based on a wireless, batteryless, passive sensor implanted in the right pulmonary artery, which can be complemented with a kit of three peripheral devices (a pulse-oximeter, a weight scale, and a blood pressure cuff). The implant (element 1, Figure 3) measures PAP using a resonant circuit, as with that of the CardioMEMS™ HF System (see above). Implantation follows a right femoral venous access with a 14 Fr introducer; once the region of interest (i.e., the downturn landmark, vessel diameter of 12–26 mm) has been identified with a pulmonary angiography, a custom delivery system with the preloaded implant is introduced. The implant is advanced under fluoroscopic guidance, and rotated before deployment thanks to the torque handle of the delivery system. Adequate angulation ensures that the asymmetric nitinol anchors maintain the implant in the downturn, opposed to the arterial wall, promoting endothelialization. This positioning also enables anterior chest readings with a small handheld reader, which interrogates the sensor via radiofrequency.

The implant body is a glass enclosure of 4 mm × 2 mm × 20 mm, containing a capacitive microelectromechanical system pressure transducer and a small inductor coil forming a resonant circuit. The enclosure is attached to self-aligning nitinol anchors that ensure mechanical fixation within the vessel lumen.

Patients using the device are prompted by the myCordella™ Tablet to take their daily 18-second-long PAP measurement, while seated, using the handheld reader. Additional parameters (i.e., blood pressure, HR, weight, and/or oxygen saturations) may also be acquired using the Bluetooth-connected peripherals. Collected data are transferred via internet to a cloud-based portal for the clinician to review [44].

In the first in-human SIRONA study (NCT03375710, *n* = 15, 53% HFpEF/HFmrEF, 47% HFrEF), the Cordella™ System (including the PAP sensor and peripherals) demonstrated successful implantation, an absence of device-related complications at 1 month post-implantation, high adherence, and accurate PAP measurements (compared to those of catheters) [45]. The SIRONA2 (NCT04012944, *n* = 70, ~38.5% HFpEF/HFmrEF, ~61.5% HFrEF) expanded to multiple European centers, meeting once again the safety and efficacy primary endpoints (at 1 and 3 months post-implantation, respectively). These results were accompanied by superb adherence (94% data transmission rate), which, altogether, serves as supporting material for CE marking (pending) [46]. The subsequent PROACTIVE-HF pivotal trial (NCT04089059, *n* = 456, ~44.3% HFpEF, ~9.9% HFmrEF, ~45.8% HFrEF) confirmed safety and effectiveness in reducing mortality and HF hospitalizations (improving results from previous trials by the CardioMEMS™ HF System) [47]. Extended 1-year data of PROACTIVE-HF showed a 49% reduction in the combined endpoint of mortality and HF hospitalization [48]. These findings led to FDA approval, positioning the Cordella™ System as the second PAP-based RPM device in the U.S. market, with commercial rollout right after clearance.

The Cordella™ System presents a unique solution for a comprehensive assessment of HF progression, by adding vital sign context to a well-established hemodynamic measurement (i.e., PAP). The use of a handheld reader eases patient adherence and allows them to take seated PAP measurements, which reflects the daytime upright physiology and is thus more akin to when HF patients are active and symptomatic. A repurposed, hands-free version of the reader could also expand the usability of the PAP sensor for assessing exercise hemodynamics, and is arguably better suited for this endeavor than the CardioMEMS™ HF System [49,50].

However, it should be noted that U.S. coverage is restricted to a limited subset of HF patients and that reliance on seated PAP, while physiologically appealing, still requires validation (especially since most prior experience is based on supine measurements). While it awaits CE marking, the Cordella™ System also has to address another major issue: integration into clinical practice. To that end, an ongoing clinical trial (PROACTIVE-HF-2, NCT05934487) will evaluate a possible physician-directed patient self-management strategy, in which patients access their data and titrate diuretics according to a personalized protocol.

### 3.3. FIRE1 System, Now NORM™ System (Foundry Innovation & Research 1, Ltd., Dublin, Ireland)

The FIRE1 System (Foundry Innovation & Research 1, Ltd., Dublin, Ireland) proposes a passive, batteryless, crown-shaped sensor placed in the IVC, between the renal and hepatic veins, via a femoral venous access. The implantation procedure uses a custom delivery system, with a 16 Fr introducer sheath, that allows for advancing the implant to the target site under fluoroscopic guidance. Successful implantation is later confirmed with a cavagram. The implant (element 4, Figure 3) forms a resonant circuit, with nitinol wrapped in polymer-coated gold strands connected to a capacitor. As the vein collapses and expands with respiration and cardiac cycles, the implant changes conformation, modifying the inductance of the circuit. This change is captured by a belt-like external unit and uploaded to a cloud-based web application. Interrogation of the implant only takes ~1 min, with the patient wearing the external unit around the abdomen. Once processed, the FIRE1 System provides data of IVC area and collapsibility, without requiring calibration.

The FIRE1 System first demonstrated the potential of IVC-based monitoring through early experimentation in animals (*n* = 9, ovine) [51], progressing to larger animal cohorts (*n* = 20, ovine) and demonstrating safety and performance for up to 6 months [52]. More recently, a pair of first in-human studies, aiming to assess safety and feasibility of the device, were completed. These were FUTURE-HF (NCT04203576, *n* = 50, 4% HFpEF/HFmrEF, 96% HFrEF), a 6-month-long European trial, and FUTURE-HF2 (NCT05763407, *n* = 15, 26.7% HFpEF/HFmrEF, 73.3% HFrEF), a 3-month-long U.S. trial [53,54]. Regarding safety, the studies showed excellent implantation success, with no serious adverse events or adverse implant effects. As for feasibility, IVC measurements showed strong correlation (R^2^ = 0.98 and R^2^ = 0.99, respectively) with area measurements from contrast-enhanced computerized tomography scans, though occasional signal-related complications were noted (e.g., signal deterioration in first-generation implants, *n* = 4, and signal termination, *n* = 1).

The FIRE1 System reframes a well-established tool for HF follow-up in clinical settings: the ultrasound measurement of IVC diameter. By collecting IVC data, it leverages the pressure–volume discordance concept previously reported in the scientific literature [55]. This concept suggests that the IVC acts as a volume buffer, meaning IVC volume changes do not necessarily translate to intracardiac pressure changes until closer to HF hospitalization (when more aggressive treatment is required) [56]. By targeting this pre-pressure rise phase, the FIRE1 System may potentially anticipate HF decompensation earlier than approaches focused on filling pressures or their surrogates. In addition, the implant is compatible with other femoral access procedures, allowing interventional equipment to pass through.

While results from FUTURE-HF and FUTURE-HF2 are positive, the FIRE1 System still has to validate how changes in IVC—which may appear 90 days before HF hospitalization—should be interpreted and acted upon clinically. Specific thresholds and concrete response actions, with regards to directed medication, have still to be explored and validated. The newest device iteration, formally renamed as NORM™ System, which obtained the Breakthrough Device Designation by the FDA in 2025, proposes the use of a new mobile app for direct feedback and physician-directed self-management. This will be materialized in an upcoming pivotal clinical trial (FUTURE-HFII, NCT05763407).

## 4. Epicardial and Perivascular Devices

Epicardial and perivascular devices represent historically uncommon sub-groups and have continuously struggled to achieve widespread clinical adoption, or even significant clinical advancements, despite remaining among the few available options capable of providing high-fidelity data regarding the heart’s mechanical function.

**Figure 4:**
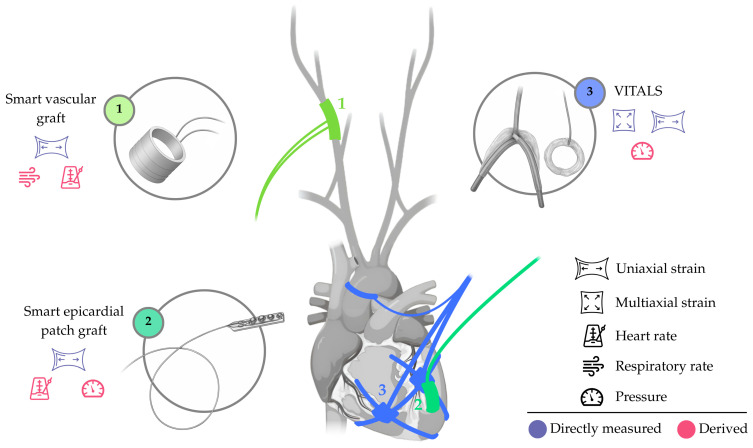
Appearance and approximate location of the sensing elements in systems designed to monitor perivascular and/or epicardial parameters. Directly measured and derived parameters are indicated. Not to scale. Only for illustrative purposes.

The limited set of technologies can be illustrated with three examples: **(i)** the VITALS, a battery-operated sensing network (element 3, Figure 4) with biventricular epicardial sensing hubs and an aortic perivascular sensor, focused on strain sensing; **(ii)** smart epicardial patch grafts, such as that developed by Z.C. Agnesi et al. [57] (element 2, Figure 4), for strain sensing on the left ventricular epicardium; and **(iii)** smart perivascular grafts, such as that developed by Z. Ma et al. [58] (element 1, Figure 4), for strain sensing at the carotid artery.

This small number of devices reflects multiple sub-group-specific challenges: for epicardial devices, factors such as stability, device size, and the requirement for open-heart procedures remain major barriers; for perivascular devices, issues such as vessel wall interaction, thrombosis risk, and durability pose similar constraints.

As a result, the theoretical applicability of these devices is currently limited to a very select population of patients, such as those already undergoing surgical intervention. However, the expectation for these sub-groups is to become more feasible and attractive as minimally invasive techniques continue to advance and technologies become more compact, biocompatible, and efficient.

## 5. Subcutaneous Devices

Insertable cardiac monitors (ICMs) form a well-established group of cardiovascular devices, historically used for the detection and management of atrial fibrillation. More recently, and drawing inspiration from cardiac implantable electronic devices (CIEDs, Section 6), ICMs have started to add new sensing functionalities (e.g., subcutaneous impedance) in an attempt to address HF. This has been made possible thanks to demonstrated efficacy in RPM for arrhythmias, advancements in battery technologies, and substantial miniaturization.

**Figure 5:**
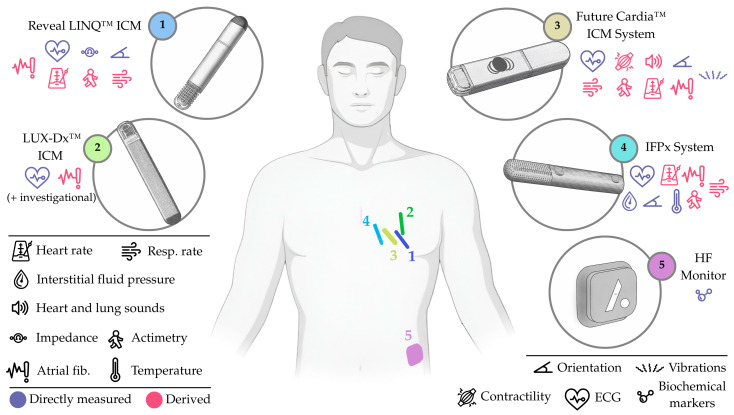
Appearance and approximate location of the sensing elements in systems designed to monitor subcutaneous parameters. Note that the HF Monitor is not fully implanted. Directly measured and derived parameters are indicated. Not to scale. Only for illustrative purposes.

Efforts towards HF monitoring have been mostly led by the Reveal LINQ™ ICM (Medtronic, Inc., Minneapolis, MN, USA; element 1, Figure 5), though other commercial ICMs for atrial fibrillation, like the Biomonitor ICM (Biotronik SE & Co., Berlin, Germany) or the Assert-IQ (Abbott, Illinois City, IL, USA), may eventually follow this path. Beyond ICMs, other devices have emerged with the original goal of supporting HF management, including the HF Monitor (element 5, Figure 5), which proposes expanding subcutaneous monitoring into the biochemical domain.

In this context, the following group of subcutaneous devices should be highlighted: **(i)** the Reveal LINQ™ ICM, a battery-operated ICM (element 1, Figure 5) for multiparametric monitoring (including subcutaneous impedance and respiratory rate); **(ii)** the LUX-Dx™ ICM, a battery-operated ICM (element 2, Figure 5), also designed for multiparametric monitoring (with most parameters under investigation); **(iii)** the Future Cardia™ ICM System, a battery-operated ICM (element 3, Figure 5), also for multiparametric monitoring, with parameters including micro-vibrations and single-lead ECG; **(iv)** the IFPx System, a battery-operated ICM with a perforated section (element 4, Figure 5), also multiparametric, but with a major emphasis on interstitial pressure monitoring (from fluid pockets); and **(v)** the HF Monitor, an abdominal, battery-operated and minimally invasive device (element 5, Figure 5) capable of measuring multiple circulating biomarkers.

Generally, these subcutaneous devices aim to occupy a middle ground between highly invasive and CIED-based options, from which only a subset of patients can benefit, and non-invasive ones, which, for the most part, depend on high patient adherence or lack evidence regarding effectiveness.

However, as devices grow distant from the cardiovascular system, the accuracy of their individual metrics declines, often necessitating the use of composite risk indices. Therefore, while most subcutaneous devices allow for ultra-high adherence and wide availability, the actionability of their composite indices, as anticipated by early experiences in CIEDs, has to be validated. In addition, practical challenges such as limited battery longevity, or the potential need for device replacement when batteries deplete, remain important considerations for most of them [59].

### 5.1. Reveal LINQ™ ICM (Medtronic, Inc., Minneapolis, MN, USA)

The Reveal LINQ™ ICM (Medtronic, Inc., Minneapolis, MN, USA) is a wireless, continuous ICM implanted over the fourth intercostal space of the left pectoral region, angled 45 degrees relative to the sternum. The insertion takes place either in the electrophysiology/catheterization lab or in-office, using a dedicated kit and supplies. The incision is less than 10 mm long, does not require conscious sedation, and is closed with sutures, staples, surgical glue, or adhesive strips [60,61]. The current commercial version of the device, for atrial fibrillation, has a battery life of 3 years. The investigational version (element 1, Figure 5), on the other hand, measures 7 × 45 × 4 mm; has two electrodes, separated 40 mm apart, for subcutaneous impedance and electrical activity measurement; a 3-axis accelerometer; and a battery (undetermined life expectancy). A portable, battery-operated, bedside monitor communicates wirelessly with the device at a daily programmed time, and transmits the data to a secure network via cellular connectivity [62].

The first in-human experience of the Reveal LINQ™ ICM was reported with the Reveal LINQ Usability trial (NCT01965899, *n* = 151), validating the ICM’s usability for arrhythmia detection with a proprietary algorithm [63]. Further studies expanded the applicability of the device to other conditions, such as high stroke risk (REVEAL AF, NCT01727297, *n* = 446) [64]. In this expansion context, results of INTERVENE-HF (NCT02698241, *n* = 66, 100% HFrEF), a feasibility study of a nurse-implemented ambulatory management strategy with TriageHF™-enabled CIEDs (explained in Section 6) [65], motivated a series of studies for HF care. These included REEF, LINQ-HF, and ALLEVIATE-HF (NCT04452149, divided in two phases, hereinafter Phase 1 and 2).

After accuracy experiments in animals (*n* = 4, porcine), the REEF (NCT02275923, *n* = 6) confirmed that changes in subcutaneous impedance reflected changes in volume status (human dialysis data) and respiratory patterns (thoracic band-based device data). Once these parameters were validated, the cohorts of LINQ-HF (NCT02758301, *n* = 104) and ALLEVIATE-HF Phase 1 (*n* = 59) were used to develop and validate a composite LINQ-HF risk score, based on subcutaneous impedance, respiratory rate, atrial fibrillation, night HR, actimetry, and HR variability. On the validation set, the reported alert sensitivity was 68%, with a 1.5 unexplained detection rate per year, and a median warning time of 64 days before HF hospitalization [66]. ALLEVIATE-HF Phase 1 also served to evaluate the safety and feasibility of the medical intervention protocol, showing no intervention-related serious adverse events at ~1-year follow-up and successful prevention of worsening HF (symptomatic resolution in 80% of cases, symptomatic prevention in 93% of cases) [67]. These results justified progression to ALLEVIATE-HF Phase 2, aimed at further validating the algorithm and assessing intervention efficacy in a larger population, including pharmacological responses beyond diuretics (e.g., long-term guideline-directed therapy) [66,67,68].

The historical role of ICMs for the detection of arrhythmias, a common event in worsening HF, makes them already useful for HF care. The Reveal LINQ™ ICM, however, has the potential to become a less invasive, widely available alternative for HF patients. To fulfill that goal, the device’s core proposition is the collection of multiparametric data, and the production of an associated risk index. This index can also be potentially strengthened by adding sensing technologies to the device, such as optical or chemical sensors [66].

Medtronic, Inc. has introduced a unique pharmacological strategy using centralized, certified HF nurses. This approach blinds patients and clinical teams to risk alerts unless recovery or safety criteria are met. Whether it streamlines ambulatory, protocol-based medication, facilitates occasional communication with clinical teams, ensures cost-effectiveness, or reduces HF hospitalizations remains to be determined.

Furthermore, whether adding parameters (e.g., subcutaneous impedance) in the investigational device affects specifications such as battery life must be clarified. In addition, for the Reveal LINQ™ ICM, how its risk indices can be turned into actionable HF interventions in a safe, effective, and tolerable way still needs to be assessed, without depending on symptoms or hemodynamic measurements. Results of the aforementioned trial (ALLEVIATE-HF, NCT04452149) will help answer these questions.

### 5.2. LUX-Dx™ ICM (Boston Scientific Corp., Marlborough, MA, USA)

The LUX-Dx™ ICM (Boston Scientific Corp., Marlborough, MA, USA) is a wireless, continuous ICM, similar to the previously described Reveal LINQ™ ICM. It is also implanted in the left pectoral region with a dedicated incision kit, in a procedure that takes ~4 min. The current version of the device, for atrial fibrillation, has dimensions 7.2 mm × 44.8 mm × 4.0 mm, incorporates two electrodes that measure subcutaneous electrical activity, and has a 3-year battery. Differently from the Reveal LINQ™ ICM, the LUX-Dx™ ICM does not require an external monitor, as device data are transmitted via Bluetooth to the patient’s smartphone and uploaded to a cloud-based portal for processing and review [69].

The LUX-Dx™ ICM has already been deemed safe and efficacious for arrhythmia detection through a set of studies, including the LUX-Dx PERFORM trial (NCT04732728, *n* = 727) [70,71,72], and clinical experience from different European centers (*n* = 368) [69]. As for application in HF, an investigational version of the LUX-Dx™ ICM (element 2, Figure 5) is currently being used in a clinical trial (LUX-Dx TRENDS, NCT04790344), where it collects data related to different physiological parameters and HF events, aiming to further develop the ICM and its algorithms. Preliminary results (*n* = 132, 15% HFpEF, 25% HFmrEF, 60% HFrEF) report new-onset atrial fibrillation in HFpEF and HFmrEF patients, suggesting early insights into HF worsening [73].

As the use of the LUX-Dx™ ICM for HF is mostly unreported to date, its full limitations are not yet clear. Nonetheless, certain challenges can be anticipated, including the uncertain actionability of the collected parameters; the possibility that device specifications may need modification with the incorporation of new sensors; the limited battery longevity (requiring eventual device replacement); and the strong reliance on smartphone-based applications, which may hinder usability in elderly or less digitally adept patients.

### 5.3. Future Cardia™ ICM System (Future Cardia, Inc., Houston, TX, USA)

The Future Cardia™ ICM System (formerly Oracle Health, Inc., now Future Cardia, Inc., Houston, TX, USA) is a wireless, continuous ICM device, implanted in the left pectoral region. Implantation takes place in-office following a 2 min procedure, requiring a simple incision and suture. The device (element 3, Figure 5) has a 2-year longevity and combines a 3-axis piezoelectric accelerometer, for monitoring micro-vibrations due to cardiac activity (i.e., seismocardiography); a single-lead ECG, for heart rhythm; and a phonocardiogram sensor, to retrieve heart and lung sounds (i.e., acoustic vibrations). Collected multiparametric data are then sent to a smartphone via Bluetooth and then pushed to a cloud-based platform, equipped with pattern recognition (machine learning) software [74].

The Future Cardia™ ICM System underwent preclinical validation in animals (*n* = 4, porcine) with induced HF (data presented by J. Bang at THT 2023, Boston), progressing to in-human implantation and testing (*n* = 5) [75], within the context of a 6-month-long, first in-human clinical trial (FC-2022-01, NCT06167434), which is still actively enrolling. Once completed, trial results will assess safety and performance in patients with arrhythmia.

The theoretical framework of the Future Cardia™ ICM System is sustained by evidence on heart sounds as cardiac acoustic biomarkers for HF worsening [76], which differentiate the device from similar ICMs. The inclusion of multiple sensors may yield improvements in sensitivity (despite not coming from the source organ), as evidenced by experience from the MultiSENSE study (NCT01128166, see Section 6) [77].

Limitations of the Future Cardia™ ICM System include its relatively short battery life, which would require repeated replacement procedures; potential usability challenges for older or frail patients, particularly if follow-up demands digital engagement; and the limited size and duration of early clinical studies, which restrict the generalizability of current evidence. Larger studies, extended follow-up, and improvements in device longevity and usability will be essential to establish its clinical value.

## 6. Trans-Compartmental Devices

Clinical guidelines recommend the use of CIEDs, like pacemakers and implantable cardiac defibrillators, in HF patients with left ventricular dysfunction and rhythm management requirements [10,78]. Besides their primary therapeutic role, and in an attempt to exploit an unmatched location that spans across heart and lungs, CIEDs’ hardware has been refined to incorporate sensing capabilities for RPM. Because of this, CIEDs form a very unique sub-group in HF care, which we refer to as trans-compartmental, that deserves attention.

**Figure 6:**
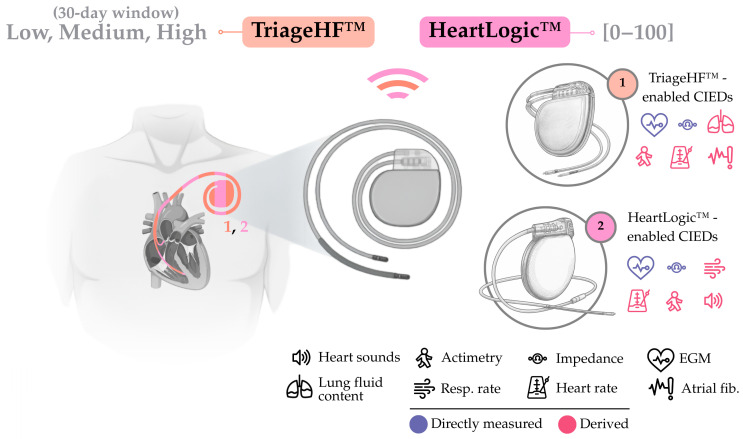
Appearance and approximate location of the sensing elements in systems that are trans-compartmental. Directly measured and derived parameters are indicated. Not to scale. Only for illustrative purposes.

CIEDs now include systems that are able to collect and monitor different variables, including thoracic impedance (i.e., between ventricular lead and generator), heart sounds, and actimetry; however, manufacturers present their proprietary integration algorithms, which generate risk scores, as the critical element for indicating HF worsening. While HeartInsight (Biotronik SE & Co., Berlin, Germany) and CorVue™ (Abbott, Illinois City, IL, USA) algorithms are remarkable examples in today’s context, the solutions by Medtronic, Inc. and Boston Scientific Corp. are the most widely used, with **(i)** the TriageHF™ being a qualitative-output algorithm and **(ii)** the HeartLogic™ being a quantitative-output algorithm, both integrating multiparametric sensing with compatible CIEDs (elements 1 and 2 in Figure 6, respectively).

A small recent study comparing TriageHF™ and HeartLogic™ showed that the former had higher sensitivity and positive predictive value, but at the cost of an increased number of alarms, while the latter had higher specificity and low false-positives, with the caveat of missing worsening episodes [79]. These findings are expected to be complemented by the ongoing REMOTI-HF trial (NCT06422832), which will evaluate the clinical impact of both algorithms.

Sensitivity, specificity, predictive values, and alarm fatigue therefore remain central performance parameters for CIED-based monitoring. Moreover, the addressable HF population is inherently limited to patients requiring a CIED in the first place. However, the most pressing challenge is the interpretation of risk scores and their integration into routine HF management, as risk scores do not correspond to directly measurable physiological parameters and are not inherently actionable. Despite this, the score-based monitoring strategy set by CIEDs has remained relevant in the RPM landscape and has been adopted and expanded by multiple other devices.

### 6.1. TriageHF™-Enabled CIEDs (Medtronic, Inc., Minneapolis, MN, USA)

TriageHF™-enabled CIEDs (Medtronic, Inc., Minneapolis, MN, USA) are based on either an implantable cardiac defibrillator or a cardiac resynchronization therapy defibrillator (element 1, Figure 6), equipped with the TriageHF™ algorithm. Collected parameters are sent via radiofrequency to a bedside home communicator, and uploaded to a cloud platform through cellular connectivity or land phone line. The algorithm integrates impedance measurements from noon to afternoon (OptiVol™ fluid index), measured every four ventricular depolarizations; night HR between midnight and morning; HR variability in 5 min windows; patient actimetry, as the number of movement minutes in a 24 h period; and HR, combining several atrial fibrillation metrics. These parameters are introduced in a Bayesian belief network that returns a risk score (low, medium, or high) [80].

The development of TriageHF™ traces back to a single-sensor approach, with the creation and implementation of the OptiVol™ (later OptiVol™ 2.0). This algorithm tracked fluid accumulation in the form of an index, dependent on (i) the magnitude and duration of thoracic impedance and (ii) the use of a nominal threshold. Even though the impedance-based approach was plausible from a theoretical perspective, results from SENSE-HF (NCT00400985, *n* = 501) indicated that the fluid index alone had very low sensitivity (20.7%) and limited predictive value for detecting HF re-hospitalizations [81]. These findings were further confirmed through OptiLink-HF (NCT00769457, *n* = 1002), as the OptiVol™ did not improve patient outcomes [82]. Overall, results from SENSE-HF motivated the development of a multiparametric algorithm, TriageHF™, capable of absorbing and strengthening the OptiVol™.

The TriageHF™ was first developed and assessed with data from implantable cardiac defibrillators and cardiac resynchronization therapy defibrillators of several clinical trials, split as development (*n* = 921) and validation (*n* = 1310) sets. When applied to the latter (using a score threshold of 20% for medium-to-high risks), the algorithm demonstrated that a high risk transition had a 46% sensitivity and 90.2% specificity in predicting HF re-hospitalization, within a 30-day window, improving the results of the OptiVol™ [83].

The TRIAGE-HF study (NCT01798797, *n* = 100) showed that high risk scores were associated with symptomatologic worsening and non-compliance with medical treatment in 83% of cases, linking the multiparametric score with real-world findings [84]. A second-generation TriageHF™, implementing OptiVol™ 2.0 (with an adaptive fluid index threshold) on implantable cardiac defibrillators and cardiac resynchronization therapy defibrillators, further demonstrated the feasibility of the algorithm, with a 47% sensitivity in predicting 30-day HF re-hospitalization and a false positive rate of 0.48 per patient-year, leading to FDA approval and CE marking [85]. The recent Triage-HF Plus trial (NCT04177199, *n* = 443) incorporated structured phone call-based assessments depending on risk scores, which allowed for a 58% reduction in HF re-hospitalizations [86]. Building on these, the post-approval study (NCT04489225) will evaluate how TriageHF™ impacts routine clinical practice.

So far, TriageHF™-enabled CIEDs have proven capable of enabling proactive interventions, which have already shown hospitalization reduction. The use of a three-level risk score (similar to traffic lights) also makes the algorithm outputs easier to understand. However, specific therapeutic guidelines are still to be implemented, as the current implementation only produces increased awareness and a closer follow-up. Future work should further assess false positives and alarm fatigue, as existing evidence remains inconsistent. To that end, strategies to reduce false positives (such as accounting on alarm duration) could also be explored [79,87].

### 6.2. HeartLogic™-Enabled CIEDs (Boston Scientific Corp., Marlborough, MA, USA)

HeartLogic™-enabled CIEDs (Boston Scientific Corp., Marlborough, MA, USA) are based on either an implantable cardiac defibrillator or a cardiac resynchronization therapy defibrillator (element 2, Figure 6), equipped with the HeartLogic™ algorithm. An external, bedside, at-home communicator receives the collected data from the CIED using radiofrequency and uploads them to a web server via Ethernet, Wi-Fi, or cellular connectivity. Data collected by CIEDs include heart sounds, respiration metrics, thoracic impedance, HR, and actimetry measurements. Thoracic impedance is measured every 2 min, heart sounds are measured every 20 min, respiration is the median rate and volume of all valid breaths, night HR is measured beat by beat between midnight and morning, and actimetry represents the number of hours a patient is active per day. Through a proprietary formula, a HeartLogic™ index is computed on a daily basis using these parameters. Index values range between 0 and 100, and are compared with baseline values (spanning up to the 3 prior months) to produce an alarm (based on a pre-set threshold increase) [88].

The MultiSENSE study (NCT01128166, *n* = 900) was the first effort in developing and validating the HeartLogic™ in cardiac resynchronization therapy defibrillators. Results from the 1-year-long study in the development cohort (*n* = 500), where clinicians and investigators were data-blinded, were used to develop the HeartLogic™ index and alert algorithm. When deployed in a test cohort (*n* = 400), and using a nominal threshold of 16, the HeartLogic™ showed 70% sensitivity, 85.7% specificity, a median alert time of 34 days before hospitalization, and a false positive rate of 1.56 per patient-year [77], which helped achieve FDA approval and CE marking in 2017.

The MultiSENSE trial also showed that HeartLogic™ indices allowed for identifying asymptomatic windows of opportunity, where HF patients could benefit from tailored management [89]. MANAGE-HF (NCT03237858), a two-phase trial initiated right after MultiSENSE, was then proposed to develop (Phase I) and evaluate (Phase II) an alert management guide based on the HeartLogic™ index. The process included a direct contact step between provider and patient, following an alert, and the implementation of a treatment algorithm to modify diuretics and guideline-directed medical therapy. Preliminary results from Phase I (*n* = 200, 100% HFrEF) showed that the early use of diuretic treatment, but not guideline-directed medical therapy shortened the alert duration, possibly indicating a gap in optimization of HF management [90].

A subsequent study on Medicare and Medicaid patients (*n* = 1548) further evaluated the algorithm’s performance in a larger set of CIEDs (including a large representation of implantable cardioverter defibrillators). The study reported a 74.5% sensitivity, an average alert time of 49 days, and a false positive rate of 1.48 per patient-year, aligned with previous findings [91]. The post-market study, PREEMPT-HF (NCT03579641, *n* = 2155), showed that the HeartLogic™ had once again a comparable performance to that previously shown, with 78.3% sensitivity, a median alert time of 35 days, and a false positive rate of 1.18 per patient-year. The study also reported that the index was significantly higher before and after re-hospitalization at 3 months post-discharge [92].

The potential of HeartLogic™-enabled CIEDs does not only come from the use of multiparametric data to deliver a holistic view, but also from the exceptional prognostic values of individual parameters. Among these, heart sounds, traditionally assessed via stethoscope but increasingly underused, stand out. In fact, data from MultiSENSE highlights that CIEDs’ heart sounds have a higher prognostic value than auscultation counterparts, which evidences their potential [93]. The quantitative nature of the HeartLogic™’s outcome also enables more complex decision-making routes. However, as for any CIED-based option, the use of HeartLogic™-enabled CIEDs is limited to a reduced group of patients.

Future work should focus on exploiting observations from PREEMPT-HF and MultiSENSE, advancing towards higher actionability. To that end, results from MANAGE-HF Phase II will help determine how index-informed care can be effectively used in the clinic.

## 7. Cutaneous and Superficial Devices

Cutaneous and superficial devices have emerged as a cornerstone of non-invasive RPM in HF. Placed directly on the skin—and, in some cases, on top of fine clothing—these devices capture vital physiological signals such as HR, respiratory rate, and thoracic impedance, offering a window into a patient’s cardiovascular status with minimal risks. Therefore, they serve as a potential alternative to highly invasive and costly counterparts, such as the V-LAP™ System or the CardioMEMS™ HF System (introduced in Section 2 and Section 3, respectively).

**Figure 7:**
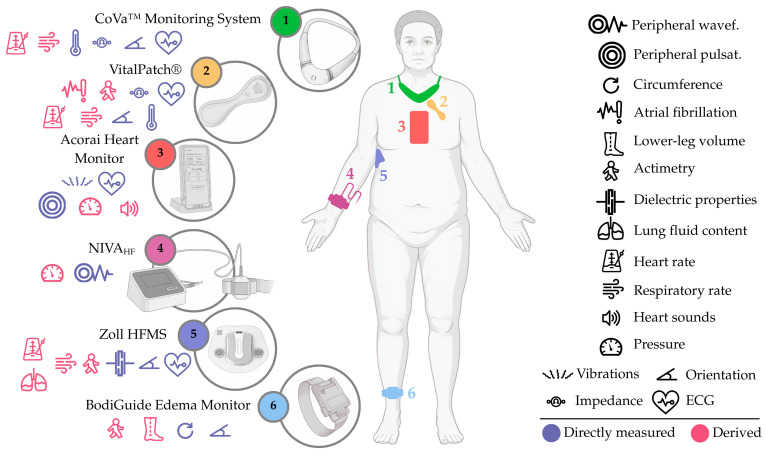
Appearance and approximate location of the sensing elements in systems designed to monitor cutaneous parameters (part 1 of 2). Directly measured and derived parameters are indicated. Not to scale. Only for illustrative purposes.

**Figure 8:**
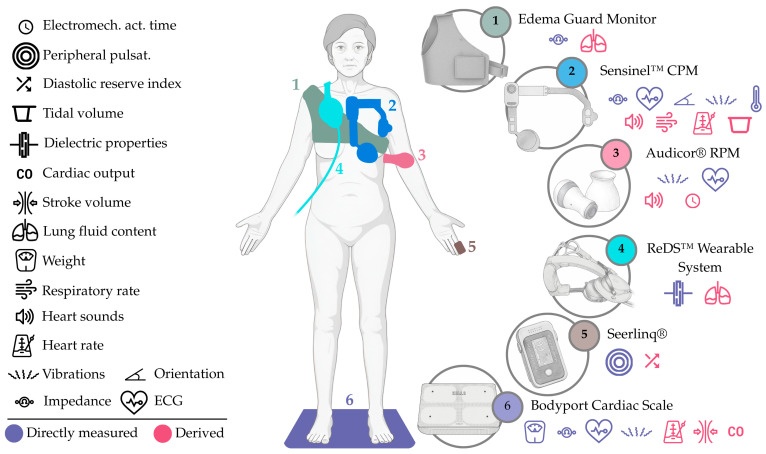
Appearance and approximate location of the sensing elements in systems designed to monitor cutaneous parameters (part 2 of 2). Note that the ReDS™ Wearable System (Sensible Medical Innovations, Ltd., Netanya, Israel) can also be used over fine clothing. Directly measured and derived parameters are indicated. Not to scale. Only for illustrative purposes.

Furthermore, advances in flexible electronics, miniaturized sensors, and wireless connectivity have made these devices increasingly comfortable and unobtrusive, enabling continuous or near-continuous monitoring, which we can recognize in multiple existing devices, including **(i)** the Zoll Heart Failure Management System (HFMS), a battery-operated adhesive patch (element 5, Figure 7) for multiparametric monitoring, with a focus on lung dielectric properties; **(ii)** the VitalPatch^®^, another battery-operated adhesive patch (element 2, Figure 7), capable of measuring multiple parameters, including thoracic impedance; **(iii)** the BodiGuide Edema Monitor, a battery-operated anklet-like device (element 6, Figure 7) for assessing ankle circumference; **(iv)** the Acorai Heart Monitor, a handheld device with redundant sensors (element 3, Figure 7) capable of deriving PAP from multiple parameters; **(v)** the NIVA_HF_, a wrist-worn device (element 4 Figure 7), intended for peripheral venous waveform assessment; **(vi)** the CoVa™ Monitoring System, a necklace-shaped device (element 1, Figure 7), capable of measuring multiple parameters, such as single-lead ECG and thoracic impedance; **(vii)** the Edema Guard Monitor, an electrode-based device (element 1, Figure 8) employing 6 electrodes and a handheld monitor, focusing on lung impedance monitoring; **(viii)** the Remote Dielectric Sensing (ReDS™) Wearable System, a wearable vest or clip (element 4, Figure 8) connected to a bedside console, capable of deriving lung fluid content from lung dielectric properties; **(ix)** the Audicor^®^ RPM, a battery-operated handheld device (element 3, Figure 8) with multiparametric sensing capabilities, including single-lead ECG and phonocardiography reading; **(x)** the Sensinel™ Cardiopulmonary Management System (CPM), a battery-operated adhesive device (element 2, Figure 8) for multiparametric monitoring, including single-lead ECG and thoracic impedance tracking; **(xi)** the Seerlinq^®^, a device based on a pulse-oximeter (element 5, Figure 8) capable of retrieving a PAP-correlated index from photoplethysmography signals; and **(xii)** the Bodyport Cardiac Scale, a battery-operated scale-like device (element 6, Figure 8), cantered around the reporting of a quantitative score from multiple parameters (e.g., single-lead ECG, impedance plethysmography, etc.).

Despite their differences, signal accuracy outside controlled settings, skin conditions, or improper placement all remain common critical challenges. Moreover, patient adherence, device durability, and seamless integration with clinical workflows continue to limit the full potential of these technologies. While cutaneous and superficial devices represent a major step toward proactive HF management, their practical impact has not fully materialized (at least, not in the same way as other invasive options); however, ongoing technological refinement and clinical validation are steadily closing this gap.

### 7.1. Zoll HFMS (Zoll Medical Corp., Chelmsford, MA, USA)

The Zoll HFMS (Zoll Medical Corp., Chelmsford, MA, USA) is based on a continuous-wear, adhesive patch with a rechargeable sensing unit, attached to the mid-axillary line. The patch (element 5, Figure 7) uses a plastic frame which accommodates the sensing unit and two ECG electrodes. It is single-use and has to be replaced every 5–7 days. The sensing unit houses a 3-axis accelerometer, a dedicated circuit, and an antenna, which assesses lung fluid content by measuring dielectric properties at radiofrequencies. The collected data are transmitted to a gateway device via Bluetooth, which uploads them to a secure cloud-based system for independent technicians to review. Results are then sent to the designated clinician.

The Zoll HFMS was validated in two clinical trials, MaTcH and ViVUS, which led to FDA clearance through the 510(k) process in 2018. ViVUS (NCT02975050, *n* = 30, unpublished results) addressed measurements like ECG, respiratory rate, and HR, while MaTcH (NCT03072732, *n* = 20) did so for lung fluid measurement, showing that it correlated well (r = 0.95) with total body fluid removal in dialysis patients [94]. A subsequent study (*n* = 120) reinforced the validity of this measurement, demonstrating good correlation with computerized tomography scans (r = 0.7) [95].

The efficacy of the Zoll HFMS has been reported in the recent BMAD trial (NCT03476187 and NCT04096040, *n* = 522, 47.3% HFpEF/HFmrEF, 51.8% HFrEF, 0.8% unknown), where patients were continuously monitored for 3 months after hospital discharge. In this case, patients on the intervention arm received directed therapy, according to weekly data reports and structured interviews, which resulted in a 38% relative risk reduction in HF hospitalization [96]. To complement these findings, another trial will be conducted to evaluate the effects of different medication responses to data, reporting their clinical impact (MAPS II, NCT05505136).

The limitations of the Zoll HFMS include regular patch replacement and its effect on long-term adherence, particularly in patients with reduced dexterity or limited support. In addition, skin irritation, possible discomfort, and the recurring cost of consumables represent further barriers that could restrict widespread adoption. On the other end, while the Zoll HFMS demands weekly patch replacements, it does not rely on daily interactions for operation or review. The semi-permanent fixation method (through the adhesive patch) also ensures measurement repeatability. Finally, the device deployment can also be easily optimized, for instance, by using it on a patient during a period of high-risk and, once stabilized, transferring it to a different patient.

### 7.2. VitalPatch^®^ (VitalConnect, Inc., San Jose, CA, USA)

The VitalPatch^®^ (VitalConnect, Inc., San Jose, CA, USA) is a battery-operated, adhesive device placed on the upper left chest, which consists of three elements: a disposable sensor patch, a disposable battery, and a reusable sensor electronics module. When the battery is inserted into the disposable patch, the electronics module activates and operates in a continuous manner. The device (element 2, Figure 7) has two electrodes facing the skin, which are used to measure single-lead ECG and thoracic impedance. Other components include a temperature sensor and a 3-axis accelerometer, which allows for deriving measurements like actimetry, orientation, HR, respiratory rate, and atrial fibrillation metrics. These measurements are sent to a paired smartphone via Bluetooth, which later uploads them, via cellular connectivity, to a secure cloud-based platform for processing with a machine learning algorithm. Patients using the VitalPatch^®^ have to replace the disposable battery every 7 days.

The accuracy of the sensing components of the VitalPatch^®^ was assessed through individual feasibility studies, including a study for ECG, HR, actimetry, and orientation (*n* = 57) [97] and another on temperature measurements (*n* = 30) [98]. By 2020, the device already obtained FDA clearance through the 510(k) process and the CE marking. Regarding HF management, the effectiveness of the VitalPatch^®^ and its algorithm were tested in the LINK-HF (NCT03037710, *n* = 100, 26% HFpEF/HFmrEF, 74% HFrEF), where the device compared the baseline measurements (at discharge) with collected data from the subsequent 3 months. The study showed that the device was able to detect HF hospitalizations in a 10-day window with a sensitivity of 76% and a specificity of 85%, with a median time between initial alert and hospitalization of 6.5 days [99].

Similarly to the Zoll HFMS, the VitalPatch^®^ allows for repeatability thanks to a semi-permanent fixation strategy; however, differently from Zoll HFMS, it has two disposable elements, which may produce an even higher economic and environmental cost in the long-term, reducing adoption. While current evidence is promising, generalizability and false positive reduction are two topics that should be directly addressed in future trials.

### 7.3. CoVa™ Monitoring System (Baxter Healthcare Corp., Deerfield, IL, USA)

The CoVa™ Monitoring System (formerly toSense, Inc., now Baxter Healthcare Corp., Deerfield, IL, USA) is a necklace-like device that attaches to the chest with single-use electrodes. The main element of the system is a sensing necklace-shaped sensor (element 1, Figure 7) that incorporates a 3-axis accelerometer and a temperature sensor. Two low-cost, disposable electrodes snap into a magnetic interface on the backside of the sensor, allowing the retrieval of single-lead ECG and thoracic impedance. Raw measurements are sent via Bluetooth to a gateway device for direct review (clinician- or patient-centred) and can also be uploaded, through cellular connectivity, to a web-based system. Once processed, other measurements can be derived from the core signals, namely HR, stroke volume, HR variability, CO, and respiratory rate. The device is designed to be worn at home, for ~5 min, 2–4 times a week.

According to data from 510(k) FDA submission of the CoVa™ Monitoring System, the device was initially assessed by means of three studies (unpublished). The first demonstrated strong correlation (r = 0.93) between thoracic impedance measurements and the volume of fluid removed in haemodialysis patients (*n* = 33). The second study (*n* = 23) confirmed findings regarding impedance when comparing the CoVa™ Monitoring System to other reference devices. The third study (*n* = 19) validated the derived respiratory rate measurements by comparing them with those of reference devices. A larger trial (NCT02719301, *n* = 85) further addressed the accuracy of the raw and derived measurements with respect to magnetic resonance imaging; however, its results remain unpublished. A pilot study on HF patients (*n* = 18) observed that thoracic impedance and derived stroke volume had potential to predict HF worsening, reporting moderate adherence (75%) (i.e., fraction of received measurements in total expected) [100]. These results were further reinforced through a subsequent study (*n* = 18), showing 70% adherence [101]. The CoVa™ Monitoring System received FDA clearance in 2017 but has not experienced wide use in clinical practice, probably due to a lack of marketing push and strong competition.

### 7.4. ReDS™ Wearable System (Sensible Medical Innovations, Ltd., Netanya, Israel)

The ReDS™ Wearable System (Sensible Medical Innovations, Ltd., Netanya, Israel) is a wearable vest or clip (element 4, Figure 8) that can be worn over light clothing and that monitors the absolute lung fluid content. The device uses two sensors, positioned at the back and front of the thorax, that emit and receive low-power electromagnetic signals. It is connected via a cable to a bedside monitor console that transmits measurements, via cellular connectivity, to a secure server, where they can be reviewed by a healthcare provider. Patients using this device take their measurements in 45 s, and can consult them in the console’s digital computer screen. The ReDS™ Wearable System already holds the CE marking and FDA clearance through the 510(k) process.

The technology behind the ReDS™ Wearable System was validated first in small preclinical and clinical studies [102], and then in a larger clinical study (*n* = 31), on decompensated and stable HF, showing high correlation between ReDS™ measurements and computerized tomography scans (ICC = 0.9) [103]. Here, values > 35% were introduced as cut-offs for pulmonary congestion, a concept that was later explored in other multiple studies. When compared to catheter measurements (*n* = 139, ~48.2% HFpEF/HFmrEF, ~51.8% HFrEF), ReDS™ values > 34% related to high filling pressures (≥18 mmHg), with a sensitivity of 90.7% and a specificity of 77.1%. However, no strong correlation was determined between the two readings, with the best concordance being observed in ambulatory patients [104]. Another study (*n* = 97) demonstrated that the 35% cut-off was only applicable for interstitial and radiographic congestion and not for sole vascular congestion [105]. In SMILE-HF (NCT02448342, *n* = 268, 29% HFpEF/HFmrEF, 71% HFpEF), the system was used for diuretic guided-therapy, where ReDS™ measurements demonstrated a 48% reduction in re-hospitalizations in the 3 months following hospital discharge [106]. In the ReDS-SAFE HF trial (NCT04305717, *n* = 100, 28% HFpEF, 12% HFmrEF, 60% HFrEF), the device proved useful for in-hospital management, as anticipated by previous findings. ReDS™-based diuretic treatment and discharge criteria using the previously introduced cut-offs were seen to reduce 30-day events (readmission, unplanned visits, death) after discharge, which was mainly attributable to a decrease in HF readmissions [107]. Building on these findings, a future ReDS-SAFE HF II trial has already been announced at THT 2025, Boston, with the aim of demonstrating that ReDS™-guided management can improve HF outcomes in a large cohort. These results will be further supported by findings of other ongoing trials (RADAR-HF, NCT03586336; NATURAL, NCT06671067; GCO 19-2678, NCT04305717), and complemented with results by nurse-led management programs (LiLAC-HF, NCT06734065).

The fundamental concept around the ReDS™ Wearable System is the assessment of dielectric properties of the thorax and their variation depending on pulmonary congestion. This type of measurement is uniquely positioned not only because of the pathophysiological insights it provides, but because it can be used on top of robes. It is also presented as a better alternative to bioimpedance, which is prone to inaccuracies caused by electrode placement, fat content, or skin moisture. Nevertheless, evidence thus far suggests the ReDS™ Wearable System could be better used as a point-of-care tool, for adequate in-hospital management and to avoid residual congestion at discharge [103,107].

### 7.5. Sensinel™ CPM (Analog Devices, Inc., Norwood, MA, USA)

The Sensinel™ CPM (Analog Devices, Inc., Norwood, MA, USA) is a wearable, battery-operated device applied to the skin of the patient’s chest, with adhesive islands following anatomical landmarks (one to the left of the sternum, one at the apex of the heart, and one in the mid-axillary line). The device (element 2, Figure 8) measures thoracic impedance, tidal volume, heart sounds, orientation, temperature, respiratory rate, HR, and single-lead ECG. A base station allows for charging the device, as well as for storing and uploading the data to a cloud-based platform, equipped with a clinician dashboard. Patients using the Sensinel™ CPM wear it for ~5 min, following two 1 min measurements, in seated and supine positions, with a 2 min rest in between.

The accuracy of the first Sensinel™ CPM version, which retrieved respiratory and congestion measurements, was evaluated in the Resp CPM Validation Study (NCT05445206, *n* = 40). Additional parameters were later introduced and assessed in independent trials (e.g., ECG validation through the ECG Validation Study, NCT05445726), leading, altogether, to its FDA clearance through the 510(k) process in 2024. More recently, the effectiveness of the different congestion measurements of the device were evaluated in the CONGEST-HF trial (NCT05026034), which was split into three cohorts. The first (*n* = 25) showed that impedance and heart sound measurements significantly correlated with weight, but not with lung ultrasound findings, in hospitalized HF patients. The second (*n* = 21) showed that impedance, but not the other two measurements, had a significant correlation with lung ultrasound findings in patients undergoing hemodialysis. The third (*n* = 20) evaluated the correlation of the third heart sound with catheter measurements but did not demonstrate a significant correlation between the two [108].

Despite providing a versatile multiparametric approach, evidence so far suggests that the Sensinel™ CPM requires further refinement and validation of its measurements, especially in clinical settings; we must find ways to strengthen its current clinical value and evaluate whether landmark-based positioning hinders adherence in real-world use. To address these, ongoing and future trials will serve to address multiple questions, including the assessment of feasibility and efficacy in different centres (multiple BETA trials, including BETA-ORLANDOHEALTH, NCT06007131; and BETA-DESERTOASIS, NCT06078280); data collection and interoperability (ADI-NYP, NCT06007079; OBS-BAPTISTJAX, NCT05980585); and even non-standard use (e.g., for pre- and post-exercise congestion) in the upcoming CONGEST-HF EX (NCT06393842).

### 7.6. Seerlinq^®^ (Seerlinq, Ltd., Trnava, Slovakia)

The Seerlinq^®^ (Seerlinq, Ltd., Trnava, Slovakia) is a non-invasive device, based on a standard pulse-oximeter coupled with a dedicated mobile app. The pulse-oximeter (element 5, Figure 8) collects photoplethysmography signals, which are transferred to a mobile phone with the Seerlinq^®^ app via Bluetooth. Signals are then uploaded to a cloud server, where they are processed and analysed using a proprietary algorithm. Patients using Seerlinq^®^ take measurements every other day, for 2 min in standing position, followed by 2 min in recumbent position. The signal change between positions provides a diastolic reserve index, inversely correlated with LVP, which is the base for RPM. A monitoring team supervises the alarms due to increased LVP and provides follow-up via phone calls. If unresolved, the patient is directed to their health provider [109].

The validation study of the Seerlinq^®^, PPG-HF (NCT06649435, *n* = 134, 55.9% HFpEF, 12% HFmrEF, 33.1% HFrEF), was divided into three cohorts. The first (*n* = 21) compared the diastolic reserve index to catheter data for the classification of normal and elevated LVP (≥15 mmHg), showing a sensitivity of 93%, a specificity of 100%, and a strong inverse correlation between parameters. The second (*n* = 112) compared it with echocardiographic data, showing a sensitivity of 85% and a specificity of 86% in the classification (where elevated LVP depended on diastolic dysfunction). The last (*n* = 9) showed that the diastolic reserve index increased following the up-titration of diuretics (i.e., decongestion) (data presented by A. Böhm at HF2025, Serbia). The technology has also showed promise for screening of HF by applying machine learning tools to photoplethysmography signals (*n* = 371) [110]. Following clinical validation, the Seerlinq^®^ received CE marking in 2025.

The idea behind Seerlinq^®^ is based on detecting changes in the hemodynamic response elicited by a positional challenge (i.e., recumbence) and capturing them in the peripheral vascular bed, which has so far proven to be a reliable surrogate for LVP. The integration of a monitoring team also adds a safety layer and reduces the workload in medical centres. Moreover, the potential application of machine learning to the collected signals suggests future avenues for automated screening and early detection of HF.

However, Seerlinq^®^ also has limitations. The diastolic reserve index, while correlated with LVP, provides an indirect estimate and may be influenced by confounding factors, such as peripheral vascular tone, autonomic variability, or measurement conditions. Additionally, current validation is limited by relatively small sample sizes in invasive comparisons and short-term follow-up, leaving questions about its long-term predictive accuracy and generalizability.

### 7.7. Bodyport Cardiac Scale (Bodyport, Inc., San Francisco, CA, USA)

The Bodyport Cardiac Scale (Bodyport, Inc., San Francisco, CA, USA) is a scale (element 6, Figure 8) embedded with ballistocardiography (i.e., for measuring vibrations and weight shifts caused by blood circulation), impedance plethysmography, and ECG sensors, which uses cellular connectivity to transmit the collected signals. Once uploaded, these are processed and presented on a web-based dashboard for clinicians to review. The dashboard is also integrated into healthcare platforms and electronic health records. From these raw signals, other measurements (like CO, HR, and stroke volume) can be derived, allowing a composite index to be obtained. Patients using the Bodyport Cardiac Scale stand bare feet on the physical platform for ~20–30 s, until they are notified to step off.

The different raw and derived measurements collected by the Bodyport Cardiac Scale were evaluated individually in a set of clinical studies, including one (*n* = 56) comparing derived cardiac parameters to direct Fick estimates for stroke volume (r = 0.81) and CO (r = 0.85) [111], and a second (*n* = 88) comparing against blood pressure (r = 0.75) [112]. These results allowed the FDA to grant clearance of the derived measurements in 2022. In a subsequent study, the Bodyport Cardiac Scale was used to monitor HF patients (*n* = 97) for 3 months and provide alarms using a fluid index with a subset of measurements (weight, impedance, and HR). The study showed that the Bodyport Cardiac Scale correctly identified 12 of the 12 HF events (100%), with a lower alert rate than conventional weight scales (15 vs. 26 alerts/100 patients/week) and a high daily adherence (86.2%) [113]. Building on this concept, SCALE-HF 1 (NCT04882449, *n* = 329, 36% HFpEF, 8% HFmrEF, 56% HFrEF) used another subset of measurements (weight and impedance) to conform a congestion index and assess HF event prediction. In this case, the index predicted 48 of the 69 HF events (70%) at 2.58 alerts per participant-year, outperforming conventional weight scales, which predicted only 24 of the 69 HF events (35%) at 4.18 alerts per participant-year [114,115].

The Bodyport Cardiac Scale demonstrates strong potential as a scalable solution exploiting the multiparametric principle, initially proposed by CIEDs. As studies to date have focused on relatively small cohorts and selected measurement subsets, further validation in larger, more heterogeneous populations is needed to confirm generalizability. The clinical decision-making pathways triggered by alerts also remain to be standardized. Finally, the portability of the device should also be considered as a limiting factor that may compromise adherence in real-world use.

## 8. Proximal Devices

**Figure 9:**
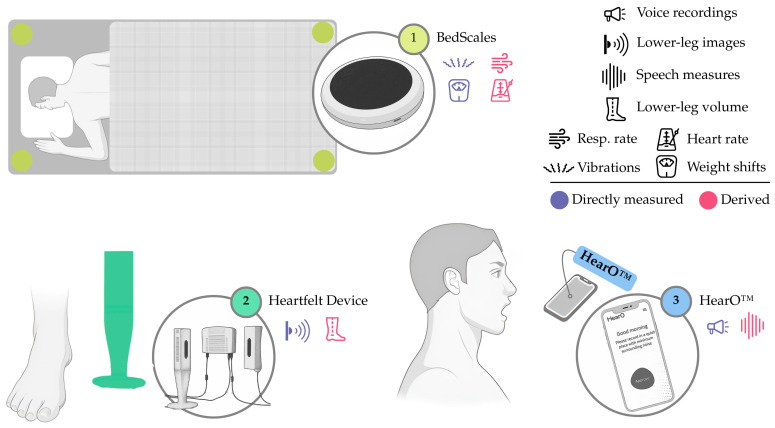
Appearance and approximate location of the sensing elements in systems designed to monitor proximal parameters. Directly measured and derived parameters are indicated. Not to scale. Only for illustrative purposes.

Even when shifting away from the bloodstream and human tissues, it is still possible to find promising device alternatives (i.e., what we call proximal devices) that blend into and operate within the background of daily life. Examples of proximal devices of the current context include **(i)** the Heartfelt Device, a mains-powered camera (element 2, Figure 9) designed to capture infra-red images of the patient’s bare foot at home; **(ii)** the BedScales, a mains-powered device composed of a four-sensor set (element 1, Figure 9) for continuous monitoring while the patient lies on a bed; and **(iii)** the HearO™, a smartphone app (element 3, Figure 9) for the continuous analysis of phonation patterns.

Propelled by historical experience using widely accessible physiological parameters [9], most of the efforts in this sub-group have been re-conducted, as exemplified by the aforementioned devices, with the aim of finding high-fidelity parameters that relate to early pathophysiological events while producing minimal patient burden.

The success of these approaches has been further reinforced recently, as some devices have even reached regulatory endorsement. The remaining question is, therefore, whether they can serve as independent solutions or will be inevitably pushed as adjuncts to more invasive technologies.

### HearO™ (Cordio Medical, Ltd., Tel Aviv, Israel)

The HearO™ (Cordio Medical, Ltd., Tel Aviv, Israel) is a smartphone app (element 3, Figure 9) that uses the patient’s mobile device to record speech data, which is adaptively processed in a cloud-based server. Patients using the app record a few sentences and establish a vocal-pattern baseline during a period of stability. This baseline is used for comparison with daily recordings of speech (six assigned sentences) that patients are prompted to record.

The first clinical validation of the HearO™ (NCT03266029, *n* = 40, 78% HFpEF/HFmrEF, 22% HFrEF) demonstrated that five speech measures where distinct between congested and decongested states, allowing for the successful separation and tagging of a set of 72 recordings, taken at discharge and admission (97.8% match) [116]. In a subsequent study (*n* = 5, 40% HFpEF, 40% HFmrEF, 20% HFrEF), the speech measures were also demonstrated to be differential between pre-dialysis and post-dialysis HF patients [117]. Since then, different trials (COMMUNITY STUDY, NCT03438799; DETECT-HF, NCT06378632) have been ongoing to refine the algorithm of HearO™. Preliminary data of the former showed that HearO™ detected HF-related events (e.g., HF hospitalizations) with a sensitivity of ~80% (versus ~35% of the traditional weight change). It was also reported that detections occurred ~3 weeks prior to the actual events (data presented at HFSA 2023, Cleveland). In addition, the HearO™ has so far shown language independence, as it has been successfully tested in Hebrew, Russian, English, and Arabic. While the HearO™ already has CE marking, it still requires additional data for FDA clearance, which will be collected in the pivotal trial, DETECT-HF [118].

The HearO™ retrieves a concept that has been previously explored in other clinical areas, such as depression or asthma, but applies it to HF. In essence, HearO™’s concept assumes that phonation patterns may be altered in distinctive ways when pulmonary congestion occurs, therefore offering a relatively low-burden, language-independent solution accordingly. However, its effectiveness depends on high patient adherence (as daily recordings are required). Furthermore, its speech metrics could presumably be influenced by factors unrelated to HF (e.g., upper respiratory infections or vocal strain), and still require large-scale validation.

## 9. Comparative Synthesis of Technical Features and Clinical Evidence

The preceding sections and sub-sections have covered each sub-group and devices in detail. However, the diversity within and between sub-groups makes direct comparison challenging. To summarize the review content and provide an initial comparative tool, we report the core technical features (e.g., sensing strategy) and latest clinical evidence of each device in Table 3.

While the table is mostly descriptive, the synthesis exercise allows for a superficial cross-comparison across taxonomic sub-groups. Additionally, the device maturity stages (properly covered in the upcoming section) are superficially hinted at by the summarized data, from which we can appreciate the very heterogeneous evidence base.

## 10. Analysis of Medical Device Readiness

The MDRL, as proposed by R.R. Seva et al. [12], integrates regulatory and standard adoption aspects to commonly used maturity scales, like the Technology Readiness Level or the Human Readiness Level. By doing so, the MDRL framework is more aligned with the med-tech industry, its development pipeline, and its regulatory nuances.

**Figure 10:**
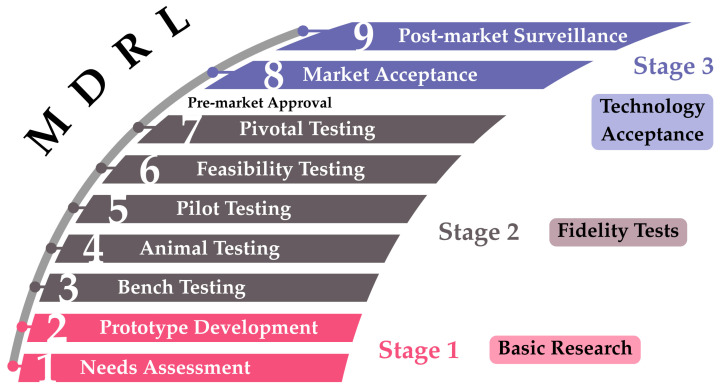
Medical Device Readiness Level (MDRL), adapted from RR. Seva et al. [12].

The MDRL is composed of nine different levels (Figure 10), ranging from Needs Assessment (MDRL 1) to Post-market Surveillance (MDRL 9), and broadly grouped within one of three stages: Basic Research (Stage 1), Fidelity Tests (Stage 2), and Technology Acceptance (Stage 3).

To objectively report the MDRL of the previously described devices, the metric was assessed independently by three investigators (I.L., A.C., and M.G.), with three-way discrepancies solved by discussion. Table 4 collects the resulting MDRL for the devices of this review. Comments on approval for commercialization are also included.

Despite not incorporating all available technologies for RPM in HF, our review shows a wide maturity spectrum in sampled devices, ranging from early experimental concepts (MDRL 3–4) to well-established commercial solutions (MDRL 8–9). The most advanced devices include invasive options, such as the CardioMEMS™ HF System and HeartLogic™-enabled CIEDs, which have demonstrated strong clinical validation; however, they also include several non-invasive ones, mostly from cutaneous and superficial sub-groups (e.g., the Bodyport Cardiac Scale, the ReDS™ Wearable System, or the VitalPatch^®^), which are now reaching near-commercial deployment. This indicates that both invasive and non-invasive strategies are capable of achieving high maturity (though the latter benefits from lower regulatory and adoption barriers). In contrast, epicardial and perivascular technologies, introduced in Section 4, remain at low readiness levels (MDRL 3–4), reflecting their early-stage status, as well as inherent technical and safety hurdles. Interestingly, some intermediate options, like those from the subcutaneous sub-group, present a more heterogeneous readiness, with no option currently impacting clinical practice.

Rather than suggesting uneven progress, these patterns likely reflect different timelines of emergence and clinical adoption across sub-groups. Taken together, these MDRLs provide an approximate overview of the current state of technological maturity across RPM solutions, from which we can appreciate both consolidated areas of progress and emerging approaches.

## 11. Proposing a Complementary Device Adoption Analysis

The dramatic increase in life expectancy over the past 25 years reflects the transformative role of medical technology in improving diagnosis and treatment.

However, a significant number of patients who survived acute events thanks to these advances now live with chronic conditions, requiring ongoing—though less intensive—care compared to the acute phase of their illness. This growing chronic population, combined with aging societies and constrained healthcare budgets, underscores the need for efficient chronic disease management as a foundation of sustainable healthcare systems.

In this context, RPM devices, such as those presented here, acquire particular importance. By enabling the continuous collection of physiological and clinical data outside the hospital, these technologies allow early detection of worsening symptoms, optimization of therapy, and personalized follow-up. RPM not only empowers patients to take an active role in managing their condition, but also reduces the need for frequent in-person visits and unplanned hospitalizations. Consequently, RPM devices represent a key strategy for improving outcomes in patients with chronic disease, as in the case of HF, while helping alleviate pressure on already overburdened healthcare systems.

Nevertheless, evaluating RPM devices for HF requires moving beyond purely technical characteristics and clinical efficacy (see Section 9 and Section 10), particularly since current device adoption largely depends on broader factors.

In this regard, we believe that a more comprehensive device assessment should also integrate two complementary evaluative dimensions, inherent to value-based healthcare: one focused on improving process efficiency (based on costs), and another on enhancing patient outcomes [119], with each dimension broken down into four metrics:
**Improving process efficiency:**a.**Device cost:** Initial acquisition and implantation expenses (if any).b.**Implementation cost:** Cost of deployment.c.**Follow-up cost:** Cost associated with ongoing monitoring.d.**Medium-term care cost:** Projected costs for continuity of care and long-term management, from a healthcare perspective (i.e., costs incurred within the healthcare sector for the estimated life expectancy).**Enhancing patient outcomes:**a.**Measurement accuracy:** Reliability in capturing clinically meaningful data (Low: 1, Medium: 2, or High: 3). For HF: based on reported correlation with gold-standards.b.**Parameter relevance:** Importance of the monitored variable for guiding therapy (Low: 1, Medium: 2, High: 3). For HF: based on existing evidence (e.g., the work by PB. Adamson [6]).c.**Enhanced follow-up:** Contribution to timely interventions and continuous monitoring (Low: 1, Medium: 2, High: 3). For HF: linked to reported (re-)hospitalization reduction.d.**Patient engagement:** Capacity to foster adherence, self-management, and activation (Low: 1, Medium: 2, High: 3. For HF: based on reported adherence or compliance.

Using this framework, the efficiency dimension can be expressed as a single cost value, while the patient outcome dimension can be merged as a score between 4 and 12 (i.e., the sum of individual metrics’ score). By doing so, devices can then be positioned in a two-axis system, providing a visual representation of their adoption potential. It should be noted, however, that cost metrics are geography-dependent, while patient outcome scores are specific to the clinical application.

Although a detailed discussion is beyond the scope of this review, we illustrate the framework with a device adoption analysis of the CardioMEMS™ HF System, reported in Table 5.

We believe these two additional dimensions provide a practical framework to fully assess the presented devices for HF monitoring, while remaining transferrable to other areas of application. Future evaluations should apply this dual perspective as a complement to more conventional, objective evaluations, thus shifting the discussion toward value generation and device adoption in today’s healthcare context.

## Figures and Tables

**Table 1:** Taxonomical dimension depending on the location of sensing element(s). Only the core sensing element(s) of a given device are considered for classification purposes.

Location of Sensing Element(s)		
**Invasive**	**Intracardiac**	Inside cardiac chambers or walls.
	**Intravascular**	Inside blood vessels.
	**Epicardial**	On the outer surface of the heart.
	**Perivascular**	On the outer surface of blood vessels, outside lumen.
	**Subcutaneous**	Beneath the skin, neither in vessels nor heart.
	**Trans-compartmental ^1^**	Spanning two or more anatomically distant compartments.
**Non-invasive**	**Cutaneous**	In contact with the skin.
	**Superficial**	On the body, but not in contact with tissues.
	**Proximal**	Near the body, completely separated from tissues.

^1^ The trans-compartmental sub-group is only applicable when sensing elements are in functionally distinct compartments (e.g., subcutaneous and intracardiac). For devices spanning closely related anatomical areas (e.g., subcutaneous and cutaneous), we use dual sub-grouping.

**Table 2:** Overview of devices included in this review (in alphabetical order at sub-group level).

Device	Sub-Group	Directly Measured Parameters	Derived Parameters
**HeartPOD^®^ (Abbott, Illinois City, IL, USA)**	Intracardiac	LAP, temperature, intracardiac EGM	-
**LV-MEMS (Abbott, Illinois City, IL, USA)**	Intracardiac	LVP	Contractility, HR, relaxation, time constant of relaxation
**PatHFinder (Synkopi, Inc., Palo Alto, CA, USA)**	Intracardiac	LAP, RAP	HR, actimetry
**Titan™ (Integrated Sensing Systems, Inc., Ypsilanti, MI, USA)**	Intracardiac	LAP or LVP	-
**V-LAP™ System (Vectorious Medical Technologies, Ltd., Tel Aviv, Israel)**	Intracardiac	LAP	-
**CardioMEMS™ HF System (Abbott, Illinois City, IL, USA)**	Intravascular	PAP	-
**Cordella™ System (Edwards Lifesciences Corp., Irvine, CA, USA)**	Intravascular	PAP, [complemented with weight, BP, HR, and SpO_2_]	-
**FIRE1 System, now NORM™ System (Foundry Innovation & Research 1, Ltd., Dublin, Ireland)**	Intravascular	IVC area	IVC collapsibility
**Smart stent (N/A)**	Intravascular	IAP	Flow changes, HR
**Smart epicardial patch graft (N/A)**	Epicardial	Uniaxial univentricular strain	HR, potentially LVP
**VITALS (N/A)**	Epicardial, Perivascular	Multiaxial biventricular strain, aortic strain	Aortic pressure
**Smart vascular graft (N/A)**	Perivascular	Carotid strain	HR, respiratory rate
**Future Cardia™ ICM System (Future Cardia, Inc., Houston, TX, USA)**	Subcutaneous	Single-lead subcutaneous ECG, orientation, vibrations (mechanical, acoustic)	Contractility, heart/lung sounds, actimetry, HR, AF, respiratory rate
**IFPx System (NXT Biomedical, LLC., Irvine, CA, USA)**	Subcutaneous	Single-lead subcutaneous ECG, IFP, temperature, orientation	Respiratory rate, AF, actimetry, HR
**LUX-Dx™ ICM (Boston Scientific Corp.,** **Marlborough, MA, USA)**	Subcutaneous	Single-lead subcutaneous ECG, additional parameters are investigational	AF
**Reveal LINQ™ ICM (Medtronic, Inc., Minneapolis, MN, USA)**	Subcutaneous	Single-lead subcutaneous ECG, subcutaneous impedance, orientation	Respiratory rate, AF, actimetry, HR
**HF Monitor (Adaptyx Biosciences, Inc., Menlo Park, CA, USA)**	Subcutaneous, Cutaneous	NT-proBNP, potassium, sodium, creatinine, urea	-
**HeartLogic™-enabled CIEDs (Boston Scientific Corp.,** **Marlborough, MA, USA)**	Trans-compartmental	Intracardiac EGM, intrathoracic impedance	Respiratory rate, HR, actimetry, heart sounds
**TriageHF™-enabled CIEDs (Medtronic, Inc., Minneapolis, MN, USA)**	Trans-compartmental	Intracardiac EGM, intrathoracic impedance	Lung fluid content, actimetry, AF, HR
**Acorai Heart Monitor (Acorai AB, Helsingborg, Sweden)**	Cutaneous	Single-lead ECG, vibrations (mechanical), peripheral arterial pulsations	Heart sounds, PAP
**Audicor^®^ Remote Patient Monitoring (Inovise Medical, Inc., Beaverton, OR, USA)**	Cutaneous	Single-lead ECG, vibrations (acoustic)	Electromechanical activation time, heart sounds
**BodiGuide Edema Monitor (BodiGuide, Inc., Bellevue, WA, USA)**	Cutaneous	Ankle circumference, orientation	Lower-leg volume, actimetry
**Bodyport Cardiac Scale (Bodyport, Inc., San Francisco, CA, USA)**	Cutaneous	Single-lead ECG, weight, impedance plethysmography, vibrations (mechanical)	HR, CO, stroke volume
**CoVa™ Monitoring System (Baxter Healthcare Corp., Deerfield, IL, USA)**	Cutaneous	Single-lead ECG, thoracic impedance, temperature, orientation	HR, respiratory rate
**Edema Guard Monitor (CardioSet Medical, Ltd., Tel Aviv, Israel)**	Cutaneous	Lung impedance	Lung fluid content
**NIVA_HF_ (VoluMetrix, LLC., Nashville, TN, USA)**	Cutaneous	Peripheral venous waveforms	PCWP
**Seerlinq^®^ (Seerlinq, Ltd., Trnava, Slovakia)**	Cutaneous	Peripheral arterial pulsations	Diastolic reserve index
**Sensinel™ Cardiopulmonary Management System (Analog Devices, Inc., Norwood, MA, USA)**	Cutaneous	Thoracic impedance, single-lead ECG, orientation, temperature, vibrations (acoustic)	Tidal volume, heart sounds, respiratory rate, HR
**VitalPatch^®^ (VitalConnect, Inc., San Jose, CA, USA)**	Cutaneous	Single-lead ECG, thoracic impedance, temperature, orientation	Actimetry, HR, respiratory rate, AF
**Zoll Heart Failure Management System (Zoll Medical Corp., Chelmsford, MA, USA)**	Cutaneous	Dielectric properties of thorax, single-lead ECG, orientation	HR, respiratory rate, actimetry, lung fluid content
**ReDS™ Wearable System (Sensible Medical Innovations, Ltd., Netanya, Israel)**	Superficial	Dielectric properties of thorax	Lung fluid content
**BedScales (Nightingale Labs Corp., San Francisco, CA, USA)**	Proximal	Weight shifts, vibrations (mechanical)	Respiratory rate, HR
**HearO™ (Cordio Medical, Ltd., Tel Aviv, Israel)**	Proximal	Voice recordings	Speech measures (five in total, not disclosed)
**Heartfelt Device (Heartfelt Technologies, Ltd., Cambridge, UK)**	Proximal	Lower-leg images	Lower-leg volume

AF, atrial fibrillation; BP, blood pressure; CIED, cardiac implantable electronic device; CO, cardiac output; ECG, electrocardiogram; EGM, electrogram; HR, heart rate; IAP, iliac artery pressure; ICM, insertable cardiac monitor; IFP, interstitial fluid pressure; IVC, inferior vena cava; LAP, left atrial pressure; LVP, left ventricular pressure; NT-proBNP, N-terminal pro B-type natriuretic peptide; PAP, pulmonary artery pressure; PCWP, pulmonary capillary wedge pressure; RAP, right atrial pressure; SpO_2_, oxygen saturation.

**Table 3:** Technical and clinical/scientific summary of devices described in this review. Research outcomes are only applicable to advancements in heart failure monitoring and/or management. Devices are ordered alphabetically at sub-group level.

Device	Taxonomic Sub-Group	Core Idea(s)	Latest In-Human Research Outcome(s)	References
**HeartPOD**^®^ **(Abbott, Illinois City, IL, USA)**	Intracardiac	LAP monitoring, complemented with signs	41% hospitalization reduction (1 year); 44% mortality reduction (2 years)	[16,19,20]
**Titan™ (Integrated Sensing Systems, Inc., Ypsilanti, MI, USA)**	Intracardiac	LVP or LAP monitoring	The device is safe and accurate	[26]
**V-LAP**™ **System (Vectorious Medical Technologies, Ltd., Tel Aviv, Israel)**	Intracardiac	LAP monitoring	The device is safe and accurate; shows potential for patient self-management	[22,23,24]
**CardioMEMS™ HF System (Abbott, Illinois City, IL, USA)**	Intravascular	PAP monitoring	28–69% hospitalization reduction (depending on geography, 1 year); 1.8% of adverse event rate	[34,35,36,37,38,39,40,41]
**Cordella™ System (Edwards Lifesciences Corp., Irvine, CA, USA)**	Intravascular	PAP monitoring, complemented with signs	49% reduction in hospitalization and mortality rate (composite, 1 year)	[47,48]
**FIRE1 System, now NORM™ System (Foundry Innovation & Research 1, Ltd., Dublin, Ireland)**	Intravascular	IVC monitoring	The device is safe and accurate	[53,54]
**Future Cardia™ ICM System (Future Cardia, Inc., Houston, TX, USA)**	Subcutaneous	Multiparametric monitoring with focus on heart sounds	-	-
**LUX-Dx** **™ ICM (Boston Scientific Corp., Marlborough, MA, USA)**	Subcutaneous	Multiparametric monitoring (details TBD)	-	-
**Reveal LINQ™ ICM (Medtronic, Inc., Minneapolis, MN, USA)**	Subcutaneous	Multiparametric monitoring	80% symptomatic resolution upon intervention (~1 year); no intervention-related adverse events; prediction of HF event with 68% sensitivity and a false positive rate of 1.5 per patient-year	[66,67]
**HeartLogic™-enabled CIEDs (Boston Scientific Corp.,** **Marlborough, MA, USA)**	Trans-compartmental	Multiparametric monitoring integrated in CIEDs (quantitative)	Prediction of HF event with 78.3% sensitivity and a false positive rate of 1.18 per patient-year	[92]
**TriageHF™-enabled CIEDs (Medtronic, Inc., Minneapolis, MN, USA)**	Trans-compartmental	Multiparametric monitoring integrated in CIEDs (qualitative)	58% re-hospitalization reduction, with 47% sensitivity, and a false positive rate of 0.48 per patient-year	[85]
**Bodyport Cardiac Scale (Bodyport, Inc., San Francisco, CA, USA)**	Cutaneous	Multiparametric monitoring with a focus on weight	Prediction of ~70% HF events and a false positive rate of 2.58 per patient-year	[114,115]
**CoVa™ Monitoring System (Baxter Healthcare Corp., Deerfield, IL, USA)**	Cutaneous	Multiparametric monitoring with a focus on thoracic impedance	-	-
**Seerlinq**^®^ **(Seerlinq, Ltd., Trnava, Slovakia)**	Cutaneous	Monitoring of PAP surrogate after hemodynamic challenge	Normal and elevated LVP predicted with 93% sensitivity and 100% specificity	Data presented at HF2025
**Sensinel™ Cardiopulmonary Management System (Analog Devices, Inc., Norwood, MA, USA)**	Cutaneous	Multiparametric monitoring with a focus on heart sounds	Thoracic impedance and heart sounds correlate with weight; thoracic impedance correlates with ultrasound findings	[108]
**VitalPatch^®^ (VitalConnect, Inc., San Jose, CA, USA)**	Cutaneous	Multiparametric monitoring with a focus on thoracic impedance	Detection of HF hospitalizations with a 76% sensitivity and 85% specificity, 10 days before event	[99]
**Zoll Heart Failure Management System (Zoll Medical Corp., Chelmsford, MA, USA)**	Cutaneous	Monitoring of thoracic dielectric properties	38% re-hospitalization reduction (3 months post-discharge)	[96]
**Remote Dielectric Sensing (ReDS™) Wearable System (Sensible Medical Innovations, Ltd., Netanya, Israel)**	Superficial	Monitoring of thoracic dielectric properties	48% re-hospitalization reduction (3 months post-discharge); no significant mortality reduction; useful for in-hospital management	[106,107]
**HearO™ (Cordio Medical, Ltd., Tel Aviv, Israel)**	Proximal	Monitoring of phonation patterns	Detection of HF hospitalizations with ~80% sensitivity, ~3 weeks before event (preliminary)	Data presented at HFSA 2023

CIED, cardiac implantable electronic device; HF, heart failure; ICM, insertable cardiac monitor; IVC, inferior vena cava; LAP, left atrial pressure; LVP, left ventricular pressure; PAP, pulmonary artery pressure.

**Table 4:** Medical Device Readiness Level (MDRL) of devices from this review (including those described in Supplementary Materials). The final MDRL score is decided by inputs from three independent investigators. Both MDRL scores and approval status are only applicable to intended uses incorporating, implicitly or explicitly, heart failure monitoring and/or management. The (C) notation alongside MDRL indicates that the device is commercialized in the U.S. and/or Europe, depending on approval (commercialization may be limited). Devices are ordered alphabetically at sub-group level.

Device	Taxonomic Sub-Group	MDRL	Approval for Commercialization	FDA Indication (Abbreviated)
**HeartPOD**^®^ **(Abbott, Illinois City, IL, USA)**	Intracardiac	6	-	-
**LV-MEMS (Abbott, Illinois City, IL, USA)**	Intracardiac	4	-	-
**PatHFinder (Synkopi, Inc., Palo Alto, CA, USA)**	Intracardiac	4	-	-
**Titan™ (Integrated Sensing Systems, Inc., Ypsilanti, MI, USA)**	Intracardiac	5	-	-
**V-LAP**™ **System (Vectorious Medical Technologies, Ltd., Tel Aviv, Israel)**	Intracardiac	6	-	-
**CardioMEMS™ HF System (Abbott, Illinois City, IL, USA)**	Intravascular	9 (C)	FDA-approved, CE-marked	Monitoring and management of HF
**Cordella™ System (Edwards Lifesciences Corp., Irvine, CA, USA)**	Intravascular	8 (C)	FDA-approved	Monitoring and management of HF
**FIRE1 System, now NORM™ System (Foundry Innovation & Research 1, Ltd., Dublin, Ireland)**	Intravascular	6	-	-
**Smart stent (N/A)**	Intravascular	4	-	-
**Smart epicardial patch graft (N/A)**	Epicardial	4	-	-
**VITALS (N/A)**	Epicardial, Perivascular	3	-	-
**Smart vascular graft (N/A)**	Perivascular	3	-	-
**Future Cardia™ ICM System (Future Cardia, Inc., Houston, TX, USA)**	Subcutaneous	5	-	-
**IFPx System (NXT Biomedical, LLC., Irvine, CA, USA)**	Subcutaneous	4	-	-
**LUX-Dx** **™ ICM (Boston Scientific Corp., Marlborough, MA, USA)**	Subcutaneous	6	-	-
**Reveal LINQ™ ICM (Medtronic, Inc., Minneapolis, MN, USA)**	Subcutaneous	7	-	-
**HF Monitor (Adaptyx Biosciences, Inc., Menlo Park, CA, USA)**	Subcutaneous, Cutaneous	3	-	-
**HeartLogic™-enabled CIEDs (Boston Scientific Corp., Marlborough, MA, USA)**	Trans-compartmental	9 (C)	FDA-approved, CE-marked	Monitoring of HF
**TriageHF™-enabled CIEDs (Medtronic, Inc., Minneapolis, MN, USA)**	Trans-compartmental	8 (C)	FDA-approved, CE-marked	Monitoring of HF
**Acorai Heart Monitor (Acorai AB, Helsingborg, Sweden)**	Cutaneous	6	-	-
**Audicor^®^ Remote Patient Monitoring (Inovise Medical, Inc., Beaverton, OR, USA)**	Cutaneous	7	-	-
**BodiGuide Edema Monitor (BodiGuide, Inc., Bellevue, WA, USA)**	Cutaneous	5	-	-
**Bodyport Cardiac Scale (Bodyport, Inc., San Francisco, CA, USA)**	Cutaneous	8 (C)	FDA-cleared	Monitoring and management of fluid-related disorders
**CoVa™ Monitoring System (Baxter Healthcare Corp., Deerfield, IL, USA)**	Cutaneous	8 (C)	FDA-cleared	Monitoring and management of fluid-related disorders
**Edema Guard Monitor (CardioSet Medical, Ltd., Tel Aviv, Israel)**	Cutaneous	7	-	-
**NIVA_HF_ (VoluMetrix, LLC., Nashville, TN, USA)**	Cutaneous	6	-	-
**Seerlinq**^®^ **(Seerlinq, Ltd., Trnava, Slovakia)**	Cutaneous	8 (C)	CE-marked	-
**Sensinel™ Cardiopulmonary Management System (Analog Devices, Inc., Norwood, MA, USA)**	Cutaneous	8 (C)	FDA-cleared	Monitoring and management of cardiopulmonary conditions
**VitalPatch^®^ (VitalConnect, Inc., San Jose, CA, USA)**	Cutaneous	8 (C)	FDA-cleared, CE-marked	Monitoring and management in general care patients
**Zoll Heart Failure Management System (Zoll Medical Corp., Chelmsford, MA, USA)**	Cutaneous	8 (C)	FDA-cleared	Monitoring and management of arrhythmias and fluid-related disorders
**Remote Dielectric Sensing (ReDS™) Wearable System (Sensible Medical Innovations, Ltd., Netanya, Israel)**	Superficial	8 (C)	FDA-cleared, CE-marked	Monitoring and management of fluid-related disorders
**BedScales (Nightingale Labs Corp., San Francisco, CA, USA)**	Proximal	6	-	-
**HearO™ (Cordio Medical, Ltd., Tel Aviv, Israel)**	Proximal	8 (C)	CE-marked	-
**Heartfelt Device (Heartfelt Technologies, Ltd., Cambridge, UK)**	Proximal	6 (C)	FDA-exempt, CE-marked	-

CE, Conformité Européenne; CIED, cardiac implantable electronic device; FDA, Food and Drug Administration; HF, heart failure; ICM, insertable cardiac monitor; MDRL, Medical Device Readiness Level.

**Table 5:** Example: Device adoption analysis of the CardioMEMS™ HF System (Abbott, Illinois City, IL, USA), with individual scores reported for each clinical adoption metric. Note that scores from the patient outcomes dimension are defined as Low (1), Medium (2), and High (3). This analysis is primarily based on data from the MONITOR-HF trial (The Netherlands, 2024), discussed by H. Mokri et al. [120]. Results are expected to be generalizable to comparable Western European healthcare systems.

Device Name	Improving Process Efficiency			Enhancing Patient Outcomes		
	Metric	Cost	Comment(s)	Metric	Score	Comment(s)
**CardioMEMS™ HF System**	**Device cost**	~EUR 10,000.00	Manufacturer prizing (The Netherlands, 2024)	**Measurement** **accuracy**	3	Correlation with catheter of |r| = 0.95 (*p* < 0.0001) [121]; Low: |r| ∈ [0.0, 0.33); Medium: |r| ∈ [0.33, 0.66); High: |r| ∈ [0.66, 1.00]
	**Implementation cost**	~EUR 2397.00	Including the cost of implantation and potential implantation complications	**Parameter** **relevance**	3	According to the work by PB. Adamson [6] regarding pulmonary and filling pressures (~25 days preceding decompensation); Low: preceding decompensation by <10 days; Medium: preceding decompensation by ≥10 days but <20 days; High: preceding decompensation by ≥20 days
	**Follow-up cost**	~EUR 631.14	Total costs for 5.72 life years, including the cost of data uploads, software updates, external unit, and possible cardiologist support	**Enhanced** **follow-up**	2	Re-hospitalization reduction of 44% [39]; Low: 0–33.3%; Medium: 33.3–66.6%; High: 66.6–100%
	**Medium-term care cost**	~EUR 62,059.86	Total associated costs for 5.72 life years, including the cost of medication (i.e., drugs and drug changes), telephone consultations, outpatient visits…	**Patient** **engagement**	3	Compliance (i.e., readings received vs. expected) of 84.3% [39]; Low: 0–33.3%; Medium: 33.3–66.6%; High: 66.6–100%
	**Total cost:** ~EUR 75,088.00			**Total score:** 11		

## Data Availability

No new data were created.

## Supplementary Materials

The following supporting information can be downloaded at https://www.mdpi.com/article/10.3390/s25206453/s1: Device descriptions not included in the main text (due to relatively low MDRL), including LV-MEMS ([122]), PatHFinder ([123]), Smart stent ([29]), VITALS ([124,125,126]), Smart vascular graft ([58]), Smart epicardial patch graft ([57]), IFPx System ([127,128,129]), Monitor HF ([130,131,132]), BodiGuide Edema Monitor ([133,134]), Acorai Heart Monitor ([135]), NIVA_HF_ ([136,137,138,139]), Edema Guard Monitor ([140,141,142,143,144]), Audicor® RPM ([145,146,147,148,149]), Heartfelt Device ([150,151,152]), BedScales ([153,154,155]).

## Abbreviations

The following abbreviations are used in this manuscript:

AF	Atrial fibrillation
BP	Blood pressure
CE	Conformité Européenne
CIED	Cardiac implantable electronic device
CO	Cardiac output
CPM	Cardiopulmonary Management System
ECG	Electrocardiogram
EGM	Electrogram
FDA	Food and Drug Administration
HF	Heart failure
HFmrEF	Heart failure with mildly reduced ejection fraction
HFMS	Heart Failure Management System
HFpEF	Heart failure with preserved ejection fraction
HFrEF	Heart failure with reduced ejection fraction
HR	Heart rate
IAP	Iliac artery pressure
ICM	Insertable cardiac monitor
IFP	Interstitial fluid pressure
IVC	Inferior vena cava
LAP	Left atrial pressure
LVP	Left ventricular pressure
MDRL	Medical Device Readiness Level
NT-proBNP	N-terminal pro B-type natriuretic peptide
PAP	Pulmonary artery pressure
PCWP	Pulmonary capillary wedge pressure
RAP	Right atrial pressure
RPM	Remote patient monitoring
SpO_2_	Oxygen saturation
