# Supplementary material for: Invasive and Non-Invasive Remote Patient Monitoring Devices for Heart Failure: A Comparative Review of Technical Maturity and Clinical Readiness
Source: Sensors (Basel). 2025 Oct 18;25(20):6453. doi: 10.3390/s25206453 (PMC12568234; doi:10.3390/s25206453)
Supplement: Supplementary file 1 [file sensors-25-06453-s001.zip › sensors-3875618-supplementary.pdf]

Supplementary Materials

Invasive and Non-Invasive Remote Patient Monitoring Devices for Heart Failure: A Comparative Review of Technical Maturity and Clinical Readiness

Ivan Luque [1], Mar Gadea [1], Anna Comas [1], Laura Becerra-Fajardo [1], Javier Colás [1,2,3], and Antoni Ivorra [1,4,*]

[1] Biomedical Electronics Research Group, Department of Engineering, Universitat Pompeu Fabra, 08018 Barcelona, Spain; ivan.luque@upf.edu (I.L.); mar.gadea@upf.edu (M.G.); anna.comas@upf.edu (A.C.); laura.becerra@upf.edu (L.B.-F.); jcolas@valorensalud.es (J.C.)
[2] Institute for Healthcare Management, Business and Law School, Esade Ramon Llull University, 08034 Barcelona, Spain
[3] Cluster for Technological Innovation and Talent in Biomedical Technologies and Biotechnology of the Community of Madrid, 28003 Madrid, Spain
[4] Serra Húnter Fellow Programme, Department of Engineering, Universitat Pompeu Fabra, 08018 Barcelona, Spain
* Correspondence: antoni.ivorra@upf.edu

S1. Introduction

S1.1. Scope

Here, we describe in detail those sampled devices with a relatively low Medical Device Readiness Level (MDRL) within their respective traditional group (i.e., invasive or non-invasive). The inclusion threshold in our main work is an MDRL > 5 (included) and an MDRL > 8 (included) for invasive and non-invasive devices, respectively. Device descriptions from Supplementary Materials will have the same distribution and focus as those of the main text.

S2. Intracardiac devices

S2.0. List of devices

The devices described here are LV-MEMS (Abbott, Illinois City, IL, USA) and PatHFinder (Synkopi, Inc., Palo Alto, CA, USA).

S2.1. LV-MEMS (Abbott, Illinois City, IL, USA)

The LV-MEMS (formerly St. Jude Medical, Inc., now Abbott, Illinois City, IL, USA) employs a wireless, batteryless sensor implanted in the apex of the left ventricle, following a right carotidal access with a 6 Fr introducer guiding sheath. The procedure involves a custom push rod with a loaded implant at the tip, so that, when placed against the cardiac tissue, it can be screwed with three clockwise rotations. The implant is composed of a cylindrical structure of 13.9 × 1.6 mm and an anchor coil—inspired by anchors from pacemaker probes—of 3 mm in length. The sensing revolves around a microelectromechanical pressure sensor, whose signals are transmitted via radiofrequency. These signals are collected by a handheld antenna, placed on the thorax, and connected to a monitor. The averaged left ventricular pressure (LVP) data is processed to derive additional functional parameters, including contractility, heart rate (HR), and relaxation metrics.

The system was firstly introduced in the context of its first animal study (n = 5, canine), where heart failure (HF) was induced by fast ventricular pacing [122]. The LV-MEMS was implanted for 35 days and interrogated in discrete time points (days 0, 21, and 35) with an antenna and recording system, developed by the CardioMEMS™ HF System (described in Section 3 of the main document). The placement and usage of the LV-MEMS was well tolerated for the duration of the study, with no apparent dislocation nor pathological response. When compared to catheter data, the LV-MEMS showed comparable performance in detecting hemodynamic changes, including HR, LVP, contractility, relaxation, and time constant of relaxation, with minimal differences observed only in LVP.

This first animal study showed that the LV-MEMS was functional and feasible, with great potential for assessing left ventricular performance, beyond filling pressures. The system would also be a great alternative to pulmonary artery pressure (PAP)-centered counterparts, as it would dismiss confounders leading to PAP increase (e.g., related to pulmonary conditions) and anticipate functional worsening earlier (as observed in the animal study). Despite promise, current evidence regarding the LV-MEMS is limited and does not support long-term safety in a large population. Considering its approach to implantation, the benefit–risk ratio should also be carefully addressed, with additional considerations for specific anticoagulation protocols and/or endothelialisation blockage.

S2.2. PatHFinder (Synkopi, Inc., Palo Alto, CA, USA)

The PatHFinder (Synkopi, Inc., Palo Alto, CA, USA) is a device based on a battery-powered, continuous, wireless implant located in the interatrial septum. Implantation follows a venous femoral access and requires the use of a custom sheath and dilator. The implant is advanced through the catheter under intracardiac echocardiography guidance, implanted through transeptal puncture, and secured to the septal wall thanks to bilateral retractile nitinol wings. The implant is a 22 mm long titanium cylinder of 6 mm in diameter, filled with silicone oil, housing a pressure transducer and an electronic circuit, as well as a battery with an intended life of 10 years.

The PatHFinder measures left atrial pressure (LAP), right atrial pressure (RAP), HR, and actimetry. The implant is envisioned to communicate with a home monitor (similar to a cell phone) which in turn uploads the data to a cloud-based portal. Such elements of the platform (i.e., the home monitor and its communication method) are still under development.

The PatHFinder has already been tested in an acute animal study (n = 2, porcine), where an implant prototype (single sensing side) was implanted and compared with catheter data (both for LAP and RAP), showing comparable performance. Further benchtop testing (in phantoms) also confirmed that the fixation system could be compatible with common right- or left-sided transeptal interventions [123].

There are two notable factors that set the PatHFinder apart from similar intracardiac devices: it provides bi-atrial information, from which additional insights, like pulmonary resistance, can be obtained and it does not require active or scheduled patient engagement, enhancing compliance. However, the implant battery's lifetime and long-term behavior are still major factors that need to be addressed.

S3. Intravascular devices

S3.0. List of devices

The devices described here are smart stents.

S3.1. Smart stents

Multiple efforts have been undertaken in recent years to develop smart stents for HF monitoring, such as the IV-Lab Device, funded by the European Innovation Council (project number: 101115545). Another notable example could be the stent developed by R. Herbert et al. [29] (hereafter referred to as Smart stent), which illustrates the potential of this technology.

The Smart stent (no company reported) is an early-stage, passive, wireless device implanted in the right iliac artery, following a left carotid access under fluoroscopic guidance. The implantable sensor consists of an inductive stent coated in parylene, composed of gold loops and polyimide connectors, with two integrated capacitive pressure sensors at both ends. The stent has a diameter of 2 mm, pre-deployment, and reaches up to 5 mm when allowed to expand. By incorporating two pressure sensors, the device can capture upstream and downstream pressures across its length, enabling the calculation of the pressure gradient and associated flow changes. An external copper loop antenna, encircling the stent, and a vector network analyzer allow for communication with the device via inductive coupling.

The Smart stent, alongside modified versions, was tested in an acute animal study (n = 1, leporine), evaluating the implantation procedure and 3-month performance in the explanted artery [29]. The study demonstrated that in-stent sensors captured pressure values and blood flow changes adequately, though it highlighted the need to refine the external unit due to low power transfer efficiency. Hence, the Smart stent requires further animal studies to monitor and achieve long-term stability, but also to finalize the details of several platform elements, including the external unit and its wireless communication method. The implantation procedure, access route, and device positioning also require refinement.

Nonetheless, the novelty of the Smart stent approach lies in its incorporation of blood flow as an additional directly measured parameter to arterial pressure, which could provide a holistic view of normal artery hemodynamics from a single device. Moreover, its design, as for every smart stent, is adaptable to different arterial sites beyond the iliac artery.

S4. Epicardial and Perivascular devices

S4.0. List of devices

The devices described here are VITALS, Smart epicardial patch graft, and smart vascular graft.

S4.1. VITALS

The VITALS (no company reported) is an early-stage device comprising a strain sensing network in the form of three elements, namely two sensing hubs, attached to the epicardium (one per ventricle) and an aortic strain sensor. The sensing hubs incorporate central electronic units spanning in two perpendicular directions through soft silicone bands, forming a cross-like, multi-axial structure. One extends from the apex to the base of a ventricle, the other perpendicularly around the wall's circumference. Each central unit includes four microelectromechanical system pressure transducers, each attached to one silicone element, which deforms in response to strain. The strain aortic sensor follows a similar logic but integrates a single pressure transducer. In its current form, the device is conceived for open-chest implantation, securing the distal ends of both sensing hubs and aortic sensor through sutures. Overall, the VITALS measures multi-axial ventricular strain and aortic circumferential strain (from which aortic pressure can be derived). Collected signals are sent to a distal circuitry, physically separated from the sensing units, following a pacemaker-type design.

Regarding evidence, the VITALS demonstrated improved fatigue resistance, a common limitation of strain-based sensors, showing durability for one million cycles (~11 days) in

benchtop testing [124]. These results motivated recent acute animal experiments (*n* = 3, porcine), where the device showed the strain's clinical applicability under a set of induced pathological states (ischemia and ventricular fibrillation). When compared to echocardiographic and catheter data, the VITALS' strain and pressure measurements showed strong agreement, with higher correlation in the former ($R^2 = 0.91$) [125].

Despite holding promise, the device still requires validation in a large, chronic animal study, which may require further design iterations to ensure long-term durability. Beyond changes to the current sensing elements, the VITALS has to define certain technological aspects, like the distal circuitry design for long-term implantation; the wireless communication protocol; and the implantation procedure, prioritizing minimal invasiveness.

The VITALS addresses a major gap in current clinical practice: the inability of techniques such as speckle-tracking echocardiography to provide continuous, remote strain monitoring. This is further motivated by evidence supporting myocardial deformation (particularly, strain) as an early indication of cardiac dysfunction, manifesting even earlier than cardiac filling pressures [126]. The integration of aortic pressure sensing further strengthens its potential to provide comprehensive cardiac assessments, positioning the VITALS as a potentially valuable tool for early HF detection and management.

S4.2. Other devices

Most devices of the perivascular and epicardial sub-groups are in early development stages, as exemplified by the VITALS. However, we find two additional early-stage devices to be worth noting, which we refer to as Smart vascular graft and Smart epicardial patch graft.

The Smart vascular graft (no company reported) is a perivascular device that incorporates a piezoresistive strain sensor to derive multiple parameters (HR, respiratory rate), and detect vascular blockages, which has been recently tested in a carotid artery (*n* = 1, leporine) [58]. The Smart epicardial patch graft (no company reported), on the other hand, is an epicardial device consisting of a sensor sutured onto the epicardium of the left ventricle. The sensor is equipped with three piezoresistive pressure sensing pillars capable of deriving HR and, potentially, LVP measurements. The sensor is physically connected to readout electronics, placed subcutaneously, and has been successfully implanted for 1 month in animals (*n* = 2, porcine) [57].

S5. Subcutaneous devices

S5.0. List of devices

The devices described here are IFPx System (NXT Biomedical, LLC., Irvine, CA, USA) and HF Monitor (Adaptyx Biosciences, Inc., Menlo Park, CA, USA).

S5.1. IFPx System (NXT Biomedical, LLC., Irvine, CA, USA)

The IFPx System (NXT Biomedical, LLC., Irvine, CA, USA) proposes a subcutaneous insertable, wireless monitor, implanted in the thorax, similarly to commercial insertable cardiac monitors (ICMs), following a 5-minute-long procedure. The IFPx System consists of a perforated capsule, with a semi-permeable membrane, a temperature-compensated pressure transducer, and an accelerometer. Once deployed, neovascularization occurs around and within the device, allowing the formation of an internal fluid pocket. The device is envisioned to collect, among others, interstitial fluid pressure, HR, actimetry, and orientation (data presented by A.M.K. Rothman at THT 2023, Boston) and upload these to a cloud-based digital health system, accessible by care providers.

The work behind the IFPx System started with an animal experiment (*n* = 1, porcine) with insertable perforated capsules. The study aimed to compare interstitial fluid pressure signals with respect to different gold-standard hemodynamic measurements (PAP, central venous pressure, and LVP), in fluid loading and fluid removal scenarios. Results from the experiment confirmed that neovascular tissue around the capsules permitted the formation of fluid pockets, whose pressure compared to hemodynamic parameters. Pockets in perforated capsules were unstable over time, due to tissue growth, which motivated the development of the current IFPx System. The first prototype was then assessed in another animal study (*n* = 10, porcine) for 3 months post-implantation, where neovascularization and long-term behavior were characterized. Here, fluid pockets were demonstrated to be stable for the whole duration of the study, as later confirmed through imaging (computerized tomography and microscopy) [127,128].

The IFPx System takes inspiration from classic work by A. Guyton based on the implantation of perforated capsules in animals, which demonstrates that interstitial pressures are typically negative under normal physiological conditions [129], offering similar parameter coverage to ICMs, with the incorporation of interstitial fluid pressure.

Despite potential, there are certain critical questions around the IFPx System that remain to be answered, including the effect on fluid pockets by long-term scar tissue overgrowth around the device, as well as the calibration, drift, and interpretation of the interstitial fluid pressure during HF monitoring.

S5.2. HF Monitor (Adaptyx Biosciences, Inc., Menlo Park, CA, USA)

The HF Monitor (Adaptyx Biosciences, Inc., Menlo Park, CA, USA) is a minimally invasive, early-stage device for continuous molecular monitoring that adheres to the abdomen, and can be worn for 10-14 days before replacement. The device uses a small, flexible probe inserted into the dermis that extracts analytes from the interstitial fluid. Inside, the device contains molecular switches, specific for different molecules, that emit fluorescent light. This fluorescence intensity, which is concentration-dependent, can be measured with a miniature optoelectric reader. Other elements of the system (such as the platform for data processing and review) have not been described to date. The HF Monitor is proposed for the continuous monitoring of different gold-standard biomarkers of HF, including natriuretic peptides; electrolytes (potassium and sodium); and kidney function indicators (creatinine and urea), which are known to precede HF symptomatic decompensation.

On that note, the first HF Monitor prototype has proven capable of measuring physiological concentrations of different molecules (glucose, potassium, lactate, and cortisol) in benchtop testing [130].

The HF Monitor is supported by a unique technology that allows to assess unconventional molecular targets. As some of the proposed analytes are direct biomarkers of pathophysiological alterations (e.g., neurohormonal activation), the HF Monitor could have a profound impact on how to direct medical therapy. This could be particularly relevant in helping to reach a more balanced diuresis, aiming for homeostatic restoration, or to optimize guideline-directed medical therapy, aiming for a more aggressive—yet safer—up-titration [131,132].

However, several potential limitations of the HF Monitor should be acknowledged. First, as with other devices based on molecular sensors, the device requires frequent replacements, which may reduce long-term convenience and patient adherence. In addition, experience with more mature technologies (e.g., continuous glucose monitors) hint at other possible challenges, including local skin irritation, problems with adhesion (e.g., in the presence of sweating), and possible erroneous readings (e.g., in extremely hot or humid environments). Finally, the interference of medication with analyte detection is a major concern that should also be addressed.

S6. Trans-compartmental devices

S6.0. List of devices

Devices described here: None.

S7. Cutaneous and Superficial devices

S7.0. List of devices

Devices described here: BodiGuide Edema Monitor (BodiGuide, Inc., Bellevue, WA, USA), Acorai Heart Monitor (Acorai AB, Helsingborg, Sweden), NIVA$_{HF}$ (VoluMetrix, LLC., Nashville, TN, USA), Edema Guard Monitor (CardioSet Medical, Ltd., Tel Aviv, Israel), and Audicor® RPM (Inovise Medical, Inc., Beaverton, OR, USA).

S7.1. BodiGuide Edema Monitor (BodiGuide, Inc., Bellevue, WA, USA)

The BodiGuide Edema Monitor (BodiGuide, Inc., Bellevue, WA, USA) is a wearable, battery-operated anklet-like device, capable of measuring ankle orientation and ankle circumference with 1 mm accuracy. From these raw data, lower-leg volume (i.e., peripheral oedema) and actimetry can be derived. The anklet is composed of a proprietary position sensor, an accelerometer, and a battery. Furthermore, the anklet device is complemented by a data gateway and a software backend (for storing, managing, and processing data). The anklet is waterproof, records measurements automatically every ~10 minutes, and has a battery life of 6 months.

The BodiGuide Edema Monitor was first described in an internal study by D. Kessler [133], followed by a feasibility pilot study (n = 12), presented in HFSA 2021, Denver, which reported that circumference measurements changed (with respect to baseline) during abrupt diet or medication changes and during HF worsening. More recently, the BodiGuide Edema Monitor was reported in a case study of a HF patient (n = 1), where anklet measurements correlated with weight [134].

The device is a promising alternative to traditional weight tracking, as it does not require high adherence. Nevertheless, no additional data, especially regarding studies on a large cohort or evaluating clinical efficacy, have been reported to date.

S7.2. Acorai Heart Monitor (Acorai AB, Helsingborg, Sweden)

The Acorai Heart Monitor (Acorai AB, Helsingborg, Sweden) is a handheld device placed on the chest, alongside the sternum. The device has four duplicated sensing technologies, which include stereo digital stethoscopes, electrocardiogram (ECG) electrodes, seismocardiography sensors, and light sensors, a duplicative strategy that ensures noise cancellation and enhances robustness. Raw signals (i.e., phonocardiography, ECG, seismocardiography, and photoplethysmography) are processed within the device (avoiding reliance on cloud processing) with a machine learning algorithm and presented to the user. By means of processing, intracardiac pressures and PAP (in mmHg) can be estimated. Recording time takes ~2 minutes while the patient is in supine position. The Acorai Heart Monitor is intended to be used as a clinical aid (i.e., to improve decision-making) within hospitals and ambulatory settings.

The Acorai Heart Monitor has been validated, to date, through two clinical studies. The results of the first (a pilot observational study conducted in Sweden) were presented at THT 2023, Boston (n = 336), and showed strong correlation (r = 0.8) between the Acorai Heart Monitor output and catheter measurements, with a sensitivity of 80% [135]. As the sample size could not eradicate overfitting, the subsequent CAPTURE-HF trial (NCT05835024, n = 1602) aimed to enlarge the

sample size and address generalization by enrolling American patients from multiple sites. Enrolment finished by the end of 2024, but results are not available yet. Furthermore, the algorithm will continue to be trained and validated through data from other future trials, such as the Pre-clinical RHC study (NCT07010562). While promising, it should be stressed that the Acorai Heart Monitor is not intended for continuous remote patient monitoring (RPM) outside the clinic, which is its main limitation.

S7.3. NIVA$_{HF}$ (VoluMetrix, LLC., Nashville, TN, USA)

The NIVA$_{HF}$ (VoluMetrix, LLC., Nashville, TN, USA) is a wrist-worn device placed over the veins of the middle of the wrist. It is based on a piezoelectric sensor, connected to a data-capture control box that detects and amplifies low-amplitude venous waveforms. With these signals, the device produces a score from the analysis of such peripheral venous waveforms (i.e., a pulmonary capillary wedge pressure estimate) using a machine learning algorithm. Patients using the device take their measurements while seated for ~2 minutes.

The first prototypes of the NIVA$_{HF}$ have undergone two relevant studies in animals and humans, which served to validate the NIVA$_{HF}$ score and its relationship with circulating blood volume. One study evaluated the score in human blood donors (*n* = 53) and controlled haemorrhage animal models (*n* = 7, porcine), showing that NIVA$_{HF}$ scores correlated with volume changes, for both haemorrhagic and blood donation scenarios, with the latter representing ~8–10% of human blood loss (500 mL), being predicted with 92% sensitivity and 84% specificity. In this case, the scores demonstrated moderate correlation (r = 0.67) with catheter measurements in animals [136]. Another study evaluated the correlation between the score and catheter measurements in humans (*n* = 83, ~28.3% with preserved or mildly reduced ejection fraction, ~71.7 with reduced ejection fraction), showing, again, a modest association (r = 0.69) and a classification of normal vs. elevated pressures (>18.0 mmHg), with a sensitivity of 80% and specificity of 53% [137]. An updated prototype device was then deployed on a larger cohort (*n* = 106, ~28.4% with preserved or mildly reduced ejection fraction, ~71.6% with reduced ejection fraction) to further train the algorithm and re-evaluate the device's clinical utility. Results showed a correlation improvement, with respect to catheter measurements, on the test set (r = 0.78) (*n* = 21) and prediction of 30-day re-hospitalizations with 91% sensitivity and 56% specificity (*n* = 84) [1380]. More recently, another study on blood donors (*n* = 33) evaluated whether NIVA$_{HF}$ could detect blood volume changes below 500 mL, thus effectively expanding upon previous work. While the device could accurately detect blood changes as low as 200 mL, the device's superior results remained at 500 mL, with no statistical significance found in volumes below this [139].

While the NIVA$_{HF}$, in its current form, is mostly intended as a point-of-care device, its assessment of peripheral volume waveforms is versatile beyond HF, as it allows for the assessment of any condition promoting hypervolemic or hypovolemic states (e.g., acute haemorrhage). Despite promise, the results thus far indicate that the device and its algorithm should be further improved to better reflect gold standards. Future work should also address how the score responds to medication changes, especially when altering congestion or vascular tone.

S7.4. Edema Guard Monitor (CardioSet Medical, Ltd., Tel Aviv, Israel)

The Edema Guard Monitor (CardioSet Medical, Ltd., Tel Aviv, Israel) is an electrode-based, impedance monitor employing six electrodes, three placed vertically on the right side of the chest, and three placed horizontally on the low edge of the right scapula, integrated into a vest. By using a six-electrode approach, the device subtracts chest wall impedance from thoracic impedance, which allows for the direct measurement of lung impedance and derivation of lung fluid content.

The electrodes' placement scheme moves them away from the large arteries to the lungs' area, enabling high-quality readings that are displayed on the handheld monitor's screen.

Regarding clinical evidence, one of the first studies of the Edema Guard Monitor explored the use of the device to predict cardiogenic pulmonary edema in hospitalized patients with diverse cardiac conditions (n = 265), showing that lung impedance correctly predicted oedema onset [140] and indicating the promise of the metric to monitor pulmonary congestion. Many years later, the device was finally studied in the HF population through IMPEDANCE-HF. In this trial (NCT01315223, n = 256), HF patients were separated in two cohorts, with one following impedance-guided medication (depending on changes from baseline), with pharmacological titration as deemed appropriate by the clinician. After 1 year follow-up, the study showed a reduction of 57% in HF hospitalizations for patients monitored with the Edema Guard Monitor.

The study observed that lung impedance started to decay 3 weeks before the HF hospitalization, a declining trend that became steeper at 2 weeks and maximal at 1 week before hospitalization [141]. The trial, which was extended for an additional year, also demonstrated that lung impedance allowed for better HF re-hospitalization prediction than traditional clinical and laboratory parameters [142].

A recent study explored the use of the Edema Guard Monitor in HF patients (n = 10) during 1 month after discharge, as well as its effects on self-care behaviour. This was based on using (and tracking) daily lung impedance and establishing daily contact (SMS, phone calls) with HF nurses, allowing on-demand medication (e.g., diuretic titration). The study showed that the use of the device was feasible, resulted in high adherence (87%), and improved patient self-care [143].

The Edema Guard Monitor is a promising alternative to haemodynamic-based devices, especially considering that it has successfully solved several common issues of impedance-based solutions, the most remarkable being the assessment of normal congestive (or dry) states, and their use as baselines [144].

Nevertheless, the device's effectiveness depends on consistent patient adherence and correct electrode placement, as the current device version requires manual electrode positioning and the vest-based version has not yet been fully detailed. Moreover, most studies have been conducted under controlled environments with close clinical supervision, raising questions about real-world applicability. Finally, the cost-effectiveness of the technology, compared with other options, remains to be determined.

S7.5. Audicor® RPM (Inovise Medical, Inc., Beaverton, OR, USA)

Audicor® RPM (Inovise Medical, Inc., Beaverton, OR, USA) is a handheld device that records electrical and acoustic signals from a single position of the chest (same location as that of the V_4 precordial lead). In particular, Audicor® RPM records single-lead ECG and phonocardiograms, and derives a series of cardiac acoustic biomarkers, the most remarkable being electromechanical activation time (i.e., the Q wave to first sound interval), the third heart sound, and the fourth heart sound. The device connects to the internet and uploads the data to a cloud-based system, which allows clinicians to visualize trends and automate alerts for HF decompensation, based on fixed, patient-specific thresholds.

Early work by P. Erne [145] helped set the fundamental acoustic biomarkers (heart sounds and electromechanical activation time) of Audicor® RPM as key markers of decompensation and cardiac dysfunction. Subsequent studies demonstrated that electromechanical activation time was a predictor of HF re-hospitalizations upon discharge (n = 45, ~62.2% with preserved or mildly reduced ejection fraction, ~37.8% with reduced ejection fraction) [146], and that the third heart sound was an independent predictor of HF mortality (n = 474) [147]. Next, results from a trial in Taiwan (NCT01298232, n = 225, 36.8% with preserved or mildly reduced ejection fraction, 63.2% with reduced ejection fraction) demonstrated that pharmacological treatment (diuretics and

guideline-directed medical therapy modifications), following Audicor® RPM metrics, allowed HF events to be reduced by 29.5% [148].

A follow-up trial on guided vs. standard treatment was announced at THT 2022, New York City, but no results have been made available to date [149]. This will hopefully serve to achieve broader validation and refine the response loop, allowing the device to reach Food and Drug Administration (FDA) clearance, especially after receiving Breakthrough Device Designation in 2021.

S8. Proximal devices

S8.0. List of devices

Devices described here: Heartfelt Device (Heartfelt Technologies, Ltd., Cambridge, UK) and BedScales (Nightingale Labs Corp., San Francisco, CA, USA).

S8.1. Heartfelt Device (Heartfelt Technologies, Ltd., Cambridge, UK)

The Heartfelt Device (Heartfelt Technologies, Ltd., Cambridge, UK) consists of a contactless, continuous, adherence-independent 3D camera, set up to capture infra-red images, that measures the lower-leg volume through computer vision and artificial intelligence software. The device is connected to household AC power in, e.g., the bedroom or living room, and captures bare-foot images in the field of view at any time of the day. The collected data are automatically sent via internet to a cloud-based, centralized monitoring service, which avoids the regular involvement of both the patient and HF team. The device has obtained Conformité Européenne marking and has FDA exempt status.

The Heartfelt Device has been shown to measure lower-leg volumes that hold a significant correlation with water displacement volumetry (r = 0.87), while being more precise [150]. When compared to Bluetooth-connected weighing scales, the device has consistently shown a higher transmission rate, as observed in the FOOT trial (NCT04072744, *n* = 26) [151] and through clinical experience (*n* = 13) [152].

While these findings are especially remarkable for a device that does not explicitly interfere with daily life, it should be noted that excessively sensitive thresholds have also been reported. This means that the current alert system could lead to alarm fatigue and/or inappropriate management. To that end, the pivotal trial (HEARTFELT, NCT06222099) will shed light onto the amount and validity of alerts, as well as their potential to improve outcomes in a large cohort of HF patients.

S8.2. BedScales (Nightingale Labs Corp., San Francisco, CA, USA)

The BedScales (Nightingale Labs Corp., San Francisco, CA, USA) is a contactless, continuous, adherence-independent sensor placed beneath the legs of a patient's home bed. The device incorporates one sensing element in every bed leg, each of which includes a force sensor and a dedicated circuit with several electronic components, encased in a rigid housing with rubber and adhesive parts. By incorporating multiple sensors, the BedScales can add and subtract measurements, accounting on patient's (re-)positioning. It also acquires chest movements during inspiration and expiration, as well as those caused by cardiac activity (i.e., ballistocardiography). By adding a time factor, the BedScales can also separate signals from different individuals sharing a bed. The digitized and amplified data from the individual force sensors is automatically transferred via micro-USB to a wall-powered central communications module, which in turn transmits it to cloud environment via Wi-Fi.

The BedScales was firstly validated by comparing its measurements with multiple references (including ECGs and chest respirometers) during sleep. In this same study, the device showed feasibility in detecting respiratory and cardiac disturbance (e.g., tachypnea) and real-world durability (3 months of continuous monitoring) in HF (n = 1) [153]. In a larger study (n = 2000), the BedScales was used to monitor respiratory rates of patients during clinical stability and clinical events (for various conditions), showing that respiration patterns allowed for 11 out of 23 hospitalizations to be alerted, including those for HF. In fact, the study identified that congestion leading to HF was one of the earliest detections, occurring at ~3 weeks before the actual hospitalization [154]. In addition, the BedScales has also been reported, more recently, to be used for the monitoring of a patient with pulmonary embolism [155].

Given that humans spend roughly one-third of their lives asleep, BedScales unlocks an exceptional opportunity to gather high-volume data with ultra-high adherence and transmission rates. Nevertheless, its performance is inherently limited to nighttime periods, making it less suitable for detecting acute events or monitoring activity-related symptoms that occur during waking hours. Moreover, the device requires a specific bed setup with compatible legs, which may reduce generalizability to different home environments. Finally, despite promising experimental data, further results are needed to demonstrate that the BedScales can adequately distinguish HF from other conditions or non-HF-related signal variations.

Abbreviations

The following abbreviations are used in the Supplementary Materials:

ECG	Electrocardiogram
FDA	Food and Drug Administration
HF	Heart failure
HR	Heart rate
ICM	Insertable cardiac monitor
LAP	Left atrial pressure
LVP	Left ventricular pressure
MDRL	Medical Device Readiness Level
PAP	Pulmonary artery pressure
RAP	Right atrial pressure
RPM	Remote patient monitoring
